# Investigating the validity of current network analysis on static conglomerate networks by protein network stratification

**DOI:** 10.1186/1471-2105-11-466

**Published:** 2010-09-16

**Authors:** Minlu Zhang, Long J Lu

**Affiliations:** 1 Division of Biomedical Informatics, Cincinnati Children's Hospital Medical Center, 3333 Burnet Avenue, Cincinnati, OH 45229, USA; 2 Department of Computer Science, University of Cincinnati, 2600 Clifton Avenue, Cincinnati, Ohio 45221, USA; 3 Department of Biomedical Engineering, University of Cincinnati, 2600 Clifton Avenue, Cincinnati, Ohio 45221, USA; 4 Department of Environmental Health, University of Cincinnati, 2600 Clifton Avenue, Cincinnati, Ohio 45221, USA

## Abstract

**Background:**

A molecular network perspective forms the foundation of systems biology. A common practice in analyzing protein-protein interaction (PPI) networks is to perform network analysis on a conglomerate network that is an assembly of all available binary interactions in a given organism from diverse data sources. Recent studies on network dynamics suggested that this approach might have ignored the dynamic nature of context-dependent molecular systems.

**Results:**

In this study, we employed a network stratification strategy to investigate the validity of the current network analysis on conglomerate PPI networks. Using the genome-scale tissue- and condition-specific proteomics data in *Arabidopsis thaliana*, we present here the first systematic investigation into this question. We stratified a conglomerate *A. thaliana *PPI network into three levels of context-dependent subnetworks. We then focused on three types of most commonly conducted network analyses, i.e., topological, functional and modular analyses, and compared the results from these network analyses on the conglomerate network and five stratified context-dependent subnetworks corresponding to specific tissues.

**Conclusions:**

We found that the results based on the conglomerate PPI network are often significantly different from those of context-dependent subnetworks corresponding to specific tissues or conditions. This conclusion depends neither on relatively arbitrary cutoffs (such as those defining network hubs or bottlenecks), nor on specific network clustering algorithms for module extraction, nor on the possible high false positive rates of binary interactions in PPI networks. We also found that our conclusions are likely to be valid in human PPI networks. Furthermore, network stratification may help resolve many controversies in current research of systems biology.

## Background

In the contemporary systems biology, the cell itself can be viewed as a complex network of interacting proteins, nucleic acids, and other biomolecules [1-6]. The network representation has been widely applied to describe various molecular systems including protein interaction maps, metabolites and reactions, transcriptional regulation maps, signal transduction pathways and functional association networks [3,7-14]. Network approaches have been proven useful in predicting protein functions, guiding large-scale experiments, facilitating drug discovery and design, and expediting novel biomarker identification [15-21].

Due to the relative scarcity of the protein-protein interaction (PPI) data, a common practice is to assemble all available binary PPIs of a certain organism into a combined static conglomerate network in order to gain a systems-level view on this organism [8,18,22,23]. Similar approaches are also applied to build other types of molecular networks, such as gene regulatory networks and metabolic networks [24,25]. These combined conglomerate networks are often an integration of diverse datasets generated from high-throughput and small-scale experiments, predictive computational methods, and expert curations [6,18,26,27]. Network analyses are then performed on these conglomerate networks. Many structural characteristics of molecular networks have been revealed [3,24,28-30].

However, since static conglomerate networks correspond to the collective of all available data components under various temporal and spatial conditions for certain organisms, studies using such networks often ignore the dynamic nature of molecular systems, and thus lose context-dependent information. Indeed, recent pioneering efforts in revealing network dynamics have suggested that the structures of static conglomerate networks might differ significantly from those of context-dependent networks under specific conditions. For example, Luscombe et al. examined the yeast transcriptional networks and discovered that topological characteristics, network motifs and crucial transcription factors as hub nodes (nodes that have high degree values), respectively, are different under five conditions (i.e., cell cycle, sporulation, diauxic shifts, DNA damage, stress response) [26]. Using the same condition-specific networks, Zhang et al. further discovered that regulatory patterns for transcription factor hubs changed substantially under different contexts [31]. Han et al. reported the status of hubs in yeast PPI network changes under different temporal conditions, thereby, can be classified into "party hubs" and "date hubs" [32]. Overlaying time-specific gene expression data onto yeast cell cycle protein interactions, de Lichtenberg et al. reported dynamic variations of protein complexes during the yeast cell cycle based on a time-dependent interaction network [33]. Recent studies have also shown that house-keeping genes and tissue-specific genes have different topological properties in the human PPI network [34,35].

**Figure 1:**
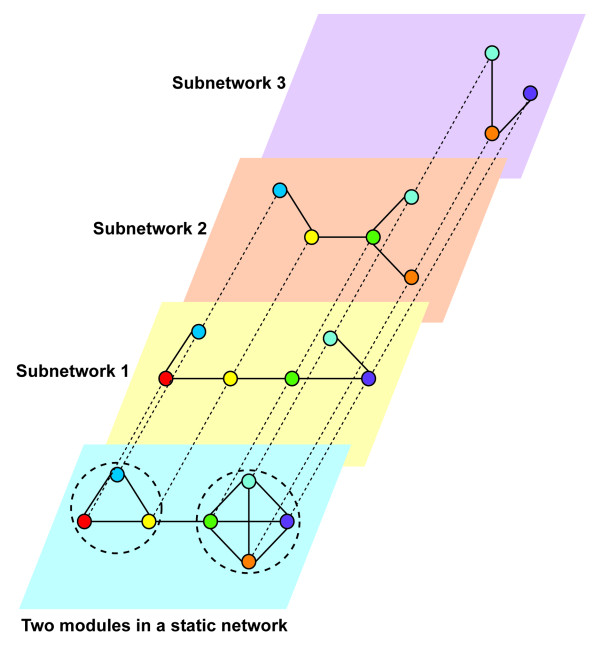
**Modules extracted from a conglomerate network may never exist in context-specific subnetworks**. In this example, a static conglomerate network containing seven nodes and ten interactions is an assembly from three context-specific subnetworks. Clearly there are two densely connected modular structures detectable by graph clustering algorithms in the conglomerate network. However, these two modules extracted do not exist under any condition-specific subnetwork, therefore representing errors in modular analysis.

Given these lines of evidence, it is reasonable to question to what degree a static conglomerate network reflects the structural and functional properties of context-dependent networks, in other words, whether the results of network analysis based on a conglomerate network still hold when taking specific biological contexts into account. For example, modules, or densely connected subnetwork structures, extracted from a static conglomerate network through graph clustering may never exist in context-dependent subnetworks because nodes and interactions are possibly context-specific, or relatively dynamic [Figure 1].

A recently published genome-scale tissue- and condition-specific proteomics data of *Arabidopsis thaliana *[36], as well as the availability of several large-scale *A. thaliana *PPI datasets has provided us with an excellent opportunity to carry out this investigation. In this study, we employed an approach called network stratification to extract context-dependent subnetworks from a large-scale conglomerate network by integrating genome-scale context-dependent data (see Methods). Particularly, conglomerate networks can be stratified in at least three dimensions: time (disparate time points or periods), space (diverse organisms, tissues, or subcellular localizations), and condition (cell cycle, stress response, DNA damage, etc.). A stratified context-dependent subnetwork, for example a PPI network containing genes that only exist in the mouse kidney or a human PPI network consisting of differentially expressed genes of primary breast cancer samples, should still encode systems-level information. Compared with unstratified conglomerate networks, stratified context-dependent subnetworks correspond to relatively homogenous contexts, and therefore network stratification may help reveal the dynamic nature of biological systems and underlying biological processes.

Using this network stratification strategy, we investigated three types of most commonly conducted network analyses: topological, functional and modular analyses [37]. Topological analysis consists of 1) various network topology characteristics such as node degree and its distribution, clustering coefficient, eccentricity, and node betweenness; 2) compatibility, or agreement of nodes and interactions between network pairs; and 3) roles of important nodes such as network hubs and bottlenecks (nodes with high betweenness centrality values). Functional analysis includes the identification of specifically enriched functions in different networks as well as differences in functional enrichment among networks. Modular analysis considers the agreement among modules or clusters extracted from different networks and functional enrichment of members in modules. The goal of this study is to address the questions of whether the results based on conglomerate networks and context-dependent subnetworks are different, and to what extent the current practice can be used. In this study, the results are mainly based on an unstratified network as well as five stratified tissue-specific subnetworks.

The novelties of our study are: First, although researchers have started to study dynamic or tissue-specific networks, the above questions have not yet been systematically addressed. Here these questions will be addressed by performing systematic comparative analyses between the unstratified network and stratified subnetworks. Second, we use proteomics data instead of mRNA expression data for integration with protein interactions, while previous works on dynamic or tissue-specific networks all used gene expression data to build subnetworks. Although there is a general trend for protein concentration to vary with mRNA expression levels, observations suggest individual gene expression intensities may not be well correlated with corresponding protein abundance values [38,39]. Therefore, it is more direct and precise to stratify protein networks with proteomics data than using gene expression data.

## Results

### Network Assembly and Subnetwork Construction

We assembled a conglomerate PPI network of *A. thaliana *by combining predicted, verified and curated PPI datasets from TAIR and AtPID databases [40,41] (see Methods). The assembled conglomerate *A. thaliana *PPI network consists of 13,136 proteins and 42,131 interactions. We then constructed 18 *A. thaliana *PPI networks with respect to specific or combinatorial temporal and spatial conditions, according to the total and 17 context-dependent protein lists of the plant described in [36] (see Methods). These 18 networks form a 4-level hierarchy [**Supp. Figure 1 in **Additional file 1]. The level-1 network is the part of the assembled conglomerate network that contains all proteins included in the proteomics data. It corresponds to the collective temporal dynamics and tissue specificity of both *A. thaliana *organs and cell cultures; henceforth referred to as the unstratified total network, or the total network. Level-2 organs and cell culture networks are coarse-stratified subnetworks representing *A. thaliana *PPIs in organs and cell culture, respectively. Level-3 tissue- and cell culture condition-specific networks are moderate-stratified subnetworks corresponding to comparatively more specific contexts. And level-4 sub-tissue-specific networks are fine-stratified subnetworks related to the most specific tissue-related contexts in this study.

Like many large-scale molecular networks, the 18 unstratified and stratified networks all display scale-free characteristics, i.e., power-law in node degree distribution [**Table S1 in **Additional file 2]. However, stratified subnetworks and the unstratified total network generally differ from one another in most network statistics including average node degree, eccentricity, and node betweenness [**Table S2 in **Additional file 2]. Generally, significant differences of average node degree values exist among 18 networks. Pairwised Welch's t-tests show that 112 out of 153 t-scores between any pair of 18 networks have p-values < 0.01, many of which are well below 1e-5 [**Table S2 in **Additional file 2]. In addition, average degrees of subnetworks are significantly different from those of randomly stratified subnetworks by node sampling that are scale-free and comparable in total nodes and interactions to the actual subnetworks (p-value < 0.01). The maximum node degree of different networks has a substantial difference intuitively, due to the fact that the unstratified total network is a union of stratified subnetworks. For instance, the protein with the maximum degree value in the unstratified total PPI network (SUMO1, or AT4G26840) has 146 interacting partners, while the highest node degree in fine-stratified cotyledons and moderate-stratified seeds PPI networks are only 41 (PBD1, or AT3G22630) and 54 (IMPA-6, or AT1G02690), respectively. Similarly, network statistics such as eccentricity and node betweenness among networks all show measurable differences with statistical significance (p-value < 0.01) [**Table S2 in **Additional file 2]. Average clustering coefficient values are relatively similar between the total network and stratified subnetworks, all being significantly higher than random expectations by node sampling (p-value < 0.01), which indicates the universal existence of modularity in all these networks. Overall, the results suggest that the majority of network statistics calculated from conglomerate networks are not transferable when evaluating context-dependent subnetworks.

**Figure 2:**
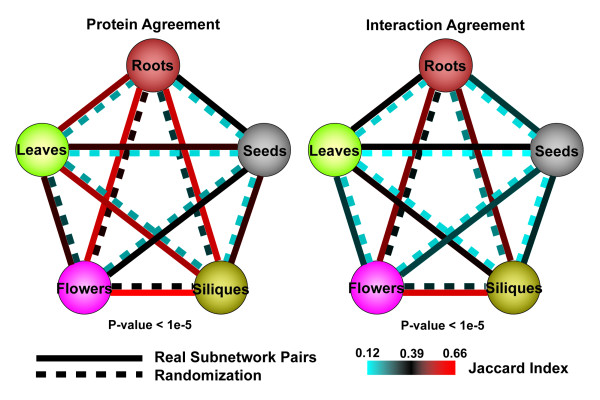
**Compatibility/Agreement between stratified subnetwork pairs**. Each circle represents a stratified tissue-specific subnetwork. For five level-3 moderately-stratified tissue-specific (roots, leaves, flowers, siliques and seeds) subnetworks, we measured network compatibility between pairs of stratified subnetworks by Jaccard Index. Node agreement values are generally higher than edge agreement values. Also shown in the figure are average compatibility scores measured by Jaccard Index for 500 randomized networks with standard deviations all less than 0.005, which are significantly different from the compatibility scores between each of the corresponding subnetwork pairs (p-value < 1e-5 for all network pairs using Student's t-test).

For the five level-3 moderate-stratified tissue-specific subnetworks (representing PPIs in roots, leaves, flowers, siliques, and seeds of *A. thaliana*), we examined network compatibility, i.e., agreement of nodes and edges between two networks, for every pair of the five subnetworks. Figure 2 shows network compatibility measured by Jaccard Index for the 10 subnetwork pairs (p-value < 1e-5 compared with randomized network compatibility; see Methods). For 10 subnetwork pairs, Jaccard Index for node agreement ranges from 0.387 to 0.655, and that for edge agreement ranges from 0.234 to 0.592 [**Table S3 in **Additional file 2]. The higher the index is, the more agreement two network share. Node agreement values are generally higher than edge agreement values. Specifically, each subnetwork contains a portion of exclusive proteins and interactions except for the seeds and siliques subnetworks, in which all nodes and interactions also exist in the flowers subnetwork. This may be due to unidentified seed- or silique-specific interactions in the conglomerate network, i.e., the incompleteness of the assembled *A. thaliana *interactome network, as well as the relatively high similarity in protein content between the flower and the silique, or the flower and the seed of the plant.

### Topology Analysis: Hubs and Bottlenecks May Change Status during Stratification

Identification of important nodes, for example, network hubs (i.e., nodes with high degree) and bottlenecks (i.e., nodes with high betweenness centrality) [30], are commonly conducted in molecular network topology analysis. If stratified PPI subnetworks differ significantly in network topology from the unstratified total network, we should expect status changes as hubs or bottlenecks for the same proteins in different networks during stratification. Such status changes may reveal dynamics of networks under specific temporal, spatial or environmental conditions. Here we focus on these two types of important nodes: network hubs and bottlenecks.

**Figure 3:**
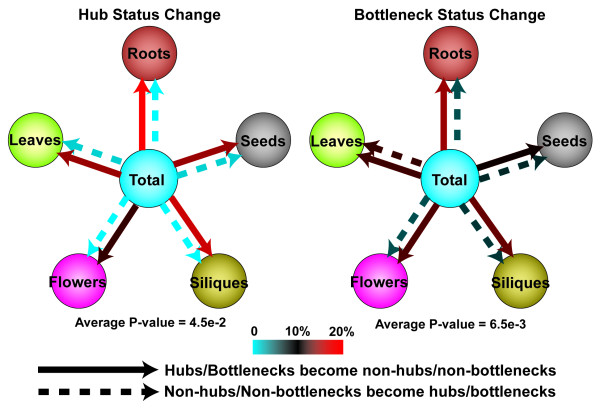
**Hub/Bottleneck status change between the total network and stratified Subnetworks**. Each circle represents a PPI network. 20% was used as the cutoff for hub/bottleneck definition. P-values were calculated by Student's t-tests comparing percentages of hub/bottleneck status change of real networks with those of randomized networks of similar size and degree distribution. The results are generally consistent when using 5% or 10% as cutoffs for hub/bottleneck definitions [**Table S3, S4 in **Additional file 2].

We found that the status of network hubs might change between the unstratified total network and stratified tissue-specific subnetworks. Specifically, hubs in the total network may change into non-hubs in stratified subnetworks. Considering nodes with the top 20% of the highest degree values in any network as hubs [30] (hub degree cutoffs are 10, 10, 8, 10, 9, 8 for total, roots, leaves, flowers, siliques and seeds subnetworks, respectively; hubs have equal or higher degree values, non-hubs are otherwise), on average, a considerable number/percentage (212/16% on average) of hubs in the total network become non-hubs in the five stratified tissue-specific subnetworks [Figure 3]. At the same time, a small number/percentage (3.2/0.8% on average; the percentage is calculated by dividing the number of non-hubs that become hubs in a subnetwork by the total number of hubs in the same subnetwork) of non-hubs in the total network become hubs in stratified subnetworks [Figure 3]. In other words, most hubs (97.4% on average) in the five stratified tissue-specific subnetworks are still hubs in the unstratified total network. The results suggest that hub analyses are transferable from a conglomerate network to context-dependent networks: most non-hubs and the majority of hubs identified in conglomerate networks are still non-hubs and hubs in context-dependent networks, respectively.

The numbers/percentages of protein hub status change, for both "hub to non-hub" and "non-hub to hub", are generally significantly different from those of randomized networks of similar sizes (p-value < 0.05) [**Table S4A in **Additional file 2]. A smaller number of hub status change compared with randomization indicates that in PPI networks, the roles of hubs and non-hubs are more likely to be preserved than those in randomized networks. This is possibly due to the evolved organizational structure in PPI networks that facilitates the preservation of hub proteins among organisms or tissues, and such structure does not exist in random networks [3]. Results showed consistency when using different percentage cutoffs (5% or 10%) for hub definition [**Table S4A and S5 in **Additional file 2].

**Figure 4:**
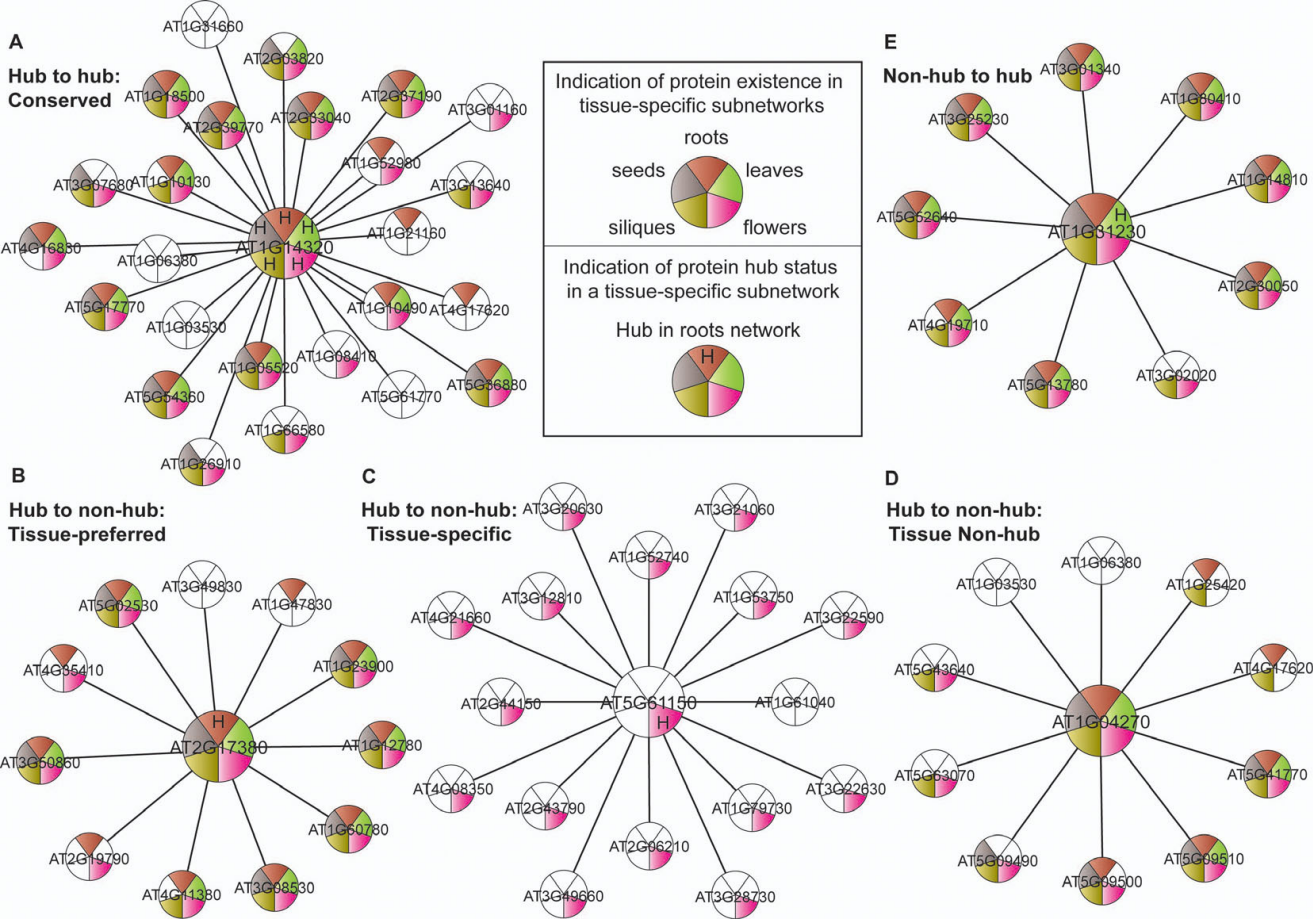
**Three scenarios of hub status change**. The center protein in each category is either a hub or a non-hub in the unstratified total network. The existence of a protein in a specific network is indicated by the color representing the network. The letter "H" over a specific color at the center protein indicates whether the protein is a hub in the corresponding tissue-specific subnetwork. A protein with no color indicates that this protein exist in the level-2 cell culture subnetwork, but not in any tissue-specific subnetwork. Three scenarios of protein hub status change (hub to hub, hub to non-hub, and non-hub to hub) include five categories, as shown in each panel.

To illustrate the biological relevance of such hub status changes, we describe three scenarios by examples in the following paragraphs, as depicted in [Figure 4].

#### Scenario 1: Hubs in the total network remain hubs in stratified subnetworks

Conserved in all five level-3 stratified tissue-specific subnetworks, SAC52 (AT1G14320, suppressor of acaulis 52) is a big hub with 25 interacting partners in the unstratified total network, which remains a hub in each of the tissue-specific subnetworks [Figure 4A]. The biological process function of SAC52, *translation*, is enriched in each of the five tissues [36,42], which explains the central role of this gene in any specific tissue. Similarly, many proteins with tissue-independently enriched functions (such as *translation*, *glycolysis*), for example GAPCP-1 (AT1G79530) and RPS8A (AT5G20290), fall in this category. Interact with many partners under various contexts, these pan-tissue hubs may include the previously defined "party hubs" that have simultaneous interactions, as well as a portion of "date hubs" that are still "date hubs" in subnetworks [32].

#### Scenario 2: Hubs in the total network become non-hubs in subnetworks

This scenario includes three sub-categories: tissue-preferred hubs, tissue-specific hubs and tissue non-hubs. Tissue-preferred hubs are those that remain hubs in at least one tissue, while losing their hub status in other tissues. For example, AP19 (protein binding/protein transporter; AT2G17380) is a hub in the total network (degree 11) [Figure 4B]. Although AP19 is still a hub in the stratified roots subnetwork (degree 10), it becomes a non-hub in stratified leaves, flowers, siliques, and seeds subnetworks (with degree of 6, 8, 6, 5, respectively). The main biological process functions of AP19 are *intracellular protein transport *(GO:0006886) and *protein transport *(GO:0015031), the latter being significantly enriched in the root of the plant [36,42]. This may explain why it becomes less important in other tissues of the plant. Proteins in this category usually have functions enriched in certain specific tissues, for example chloroplast- and cold response-related protein CPN60B (AT1G55490; hubs in the total network and stratified leaves subnetwork), thereby becoming less important and losing their hub status in other tissues.

Tissue-specific hubs are the hubs in the total network that only exist in one specific tissue and remain hubs in the tissue. VIP4 (AT5G61150; Vernalization independence 4) is a hub in the unstratified total network [Figure 4C]. When stratifying the total network, VIP4 disappears in any other tissue-specific subnetworks, yet remains a hub in stratified flowers subnetwork. The functions of VIP4 include *negative regulation of flower development *(GO:0009910) and *vernalization response *(GO:0010048) [40,43], which may explain its particular role in flowers of the plant. Although VIP4 only exists in flowers, because it is highly connected, it becomes a hub in the total network.

Tissue non-hubs lose their hub status in all tissue-specific subnetworks after stratifying the total network. Such proteins may interact with different partners and perform different functions under different contexts. For instance, RPS15 (AT1G04270; Cytosolic ribosomal protein S15) is a hub in the total network (degree 10), which becomes a non-hub in any tissue-specific subnetwork [Figure 4D]. Though not studied in this work, we also expect hubs with transient interactions (i.e., dynamic interactions that are time-dependent) to fall in this category. Because a conglomerate network combines all context-dependent interactions, the corresponding protein may appear a hub in the conglomerate network.

In this scenario, the hubs identified based on a conglomerate network are incorrect in at least one context-dependent network.

#### Scenario 3: Non-hubs in the total network are hubs in subnetworks

Appearing as a non-hub in the total network (degree 9, top 25% but not top 20%), AK-HSDH_I (AT1G31230; Aspartate kinase-homoserine dehydrogenase I) is actually a hub in the stratified leaves subnetwork (degree 8, top 20%). This non-hub to hub change, albeit not substantial in terms of degree percentage ranking, may have biological significance because the majority of its interacting partners preserve in the smaller leaves subnetwork [Figure 4E]. In fact, AK-HSDH_I have functions *chloroplast *(GO:0009507) and *chloroplast stroma *(GO:0009570) of GO biological processes, which are both leaf-related functions [44,45]. This type of hubs in a tissue-specific subnetwork is largely equivalent to the notion of "local" hubs in [35].

In order to quantitatively measure the variation of node degree, or node "hubbiness" between the unstratified total network and stratified subnetworks, we further classified nodes into five exclusive classes according to their degree values. In each network, using the top 5, 10, 20, and 50 percent of degree values as cutoffs, nodes/proteins are assigned into one of the five exclusive classes of different "hubbiness". After assigning each protein in all 18 different networks of four levels into one of the five classes, a substantial portion of proteins, 38.8% (2,562 out of 6,606) change in degree class among all networks, i.e., "hubbiness" of these nodes changes in one of the stratified subnetworks compared with that of the total network. If considering a change of two or more in degree class for the same nodes in two different networks as a "leap" change, using the same classification, 5.4% (354 out of 6,606) proteins have leap changes in their degree classes [**Table S6A in **Additional file 2]. We applied different cutoffs and different numbers of classes for classification, and the results showed consistency [**Table S7 in **Additional file 2].

**Figure 5:**
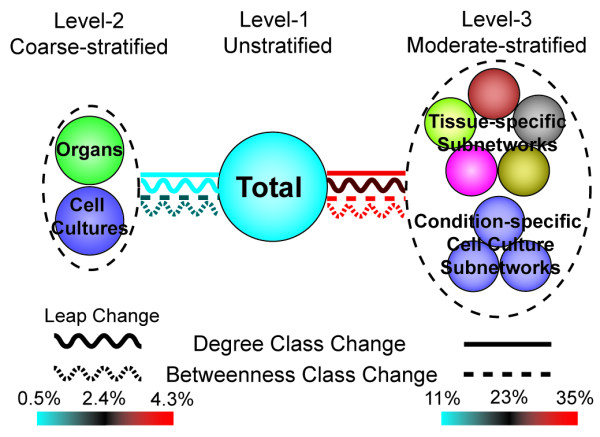
**Level-wised node degree class change**. Higher percentages of node degree/betweenness class variations exist when the total network is stratified into finer-grained subnetworks. All percentages here were calculated based on the total number of hubs/bottlenecks in the total network. A change of 2 or more in class is considered a leap change (curved lines). Level-1 versus level-2 change and leap change in both node degree and betweenness classes are smaller in percentage compared with those of level-1 versus level-3. Similarly, when only considering PPIs in different tissues but not cell culture conditions, level-1 versus level-4 change and leap change are higher in percentage than those of level-1 versus level-3 [**Table S5A in **Additional file 2].

Given the 4-level hierarchy of these 18 networks [**Supp. Figure 1 in **Additional file 1], we observed that networks from more distant levels exhibit a larger difference in node degree, i.e., node hub status change should be less observable between the level-1 total network and both level-2 coarse-stratified organs and cell culture networks than between the total network and all level-3 moderate-stratified tissue- and condition-specific subnetworks of organs and cell culture. Results show that such a trend occurs when measuring both changes and leap changes in degree classes for proteins among the total network and stratified subnetworks [Figure 5, **Table S6A in **Additional file 2].

While hubs as important nodes denote high local connectivity in networks [46], bottlenecks are nodes with high betweenness centrality that may represent connectors between clusters, or communities in networks. Among the 18 networks in the 4-level hierarchy, the overlap between network hubs and bottlenecks ranges from 0.39 to 0.55 measured by the Jaccard Index. The rather high overlap between hubs and bottlenecks is consistent with a previous study that degree and betweenness are highly correlated in PPI networks [30]. We adopted the same approach as that of hub status change measurement to measure bottleneck status change between the total network and stratified tissue-specific subnetworks. The results are similar to those of hub status change except that in contrast to the minor "non-hub to hub" changes, a higher number/percentage (9% on average, which is calculated by dividing the number of non-bottlenecks that change into bottlenecks in a subnetwork by the total number of bottlenecks in the same subnetwork) of "non-bottleneck to bottleneck" changes is observable [Figure 3, **Table S4B and Table S5 in **Additional file 2], which indicates that modules in the total network as densely connected subnetworks are relatively sensitive to the change of network topology. These results suggest that the majority of bottlenecks and non-bottlenecks in conglomerate networks remain unchanged during stratification, though bottleneck status change appears in a higher degree than hubs. Therefore, the majority of bottlenecks and non-bottlenecks identified based on conglomerate networks are still valid in context-dependent networks.

### The Total Network and Stratified Subnetworks Differ in Functional Enrichment

**Figure 6:**
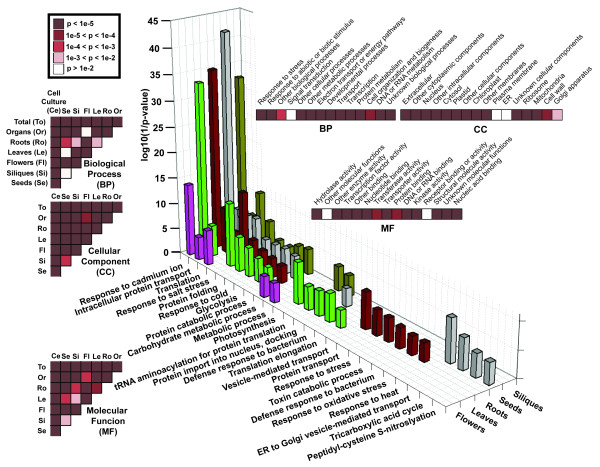
**Functional enrichment in the total network and stratified tissue-specific subnetworks using GO annotations for proteins**. Functional classification of proteins in each of the level-3 stratified tissue-specific subnetworks is evaluated using GO biological process terms. GO functional enrichment is assessed by Fisher's exact tests, and highly enriched terms with p-value < 5e-4 are shown in the figure. (Left) Different colors indicate significance of pairwise functional enrichment differences between proteins in the two networks/subnetworks, using GO slim high level terms. (Top right) Different colors indicate significance of differences in the distribution of each GO slim function term by protein annotations among networks.

Functional enrichment of proteins in networks is another commonly conducted molecular network analysis. In this section, we investigated whether the functional enrichment analysis based on the unstratified total network are consistent with that based on stratified subnetworks. We evaluated the distribution of proteins in each of the five stratified tissue-specific (roots, leaves, flowers, siliques and seeds) subnetworks into different biological processes based on the TAIR9 Gene Ontology (GO) annotations [47]. We then performed Fisher's exact tests to identify significantly enriched biological process functions in each of the stratified tissue-specific subnetworks compared with all proteins in the total network and extracted top-ranked GO functions using a significant p-value cutoff of 5e-4 [Figure 6]. House-keeping functions, such as *response to cadmium ion*, *intracellular protein transport*, *translation*, *protein folding*, and *glycolysis*, are over-represented in at least four of the five subnetworks; while tissue-specific functions are observed to be highly enriched by proteins in corresponding stratified tissue-specific subnetworks, e.g., *photosynthesis *in leaves, *vesicle-mediated transport *and *protein transport *in roots, and *response to heat *in seeds. Some of the house keeping functions, such as *intracellular protein transport*, *translation*, *protein folding*, and *glycolysis*, are also represented by human house-keeping proteins from a recent study [35]. *Response to cadmium ion *is not represented by any human house keeping protein, which is due to the difference between these two organisms.

Because a protein may have multiple GO annotations but only perform some of its annotated functions in a specific subnetwork, it would be more precise to consider interacting pairs in a network that share the same GO terms to determine their true functions in the network. Therefore, we selected interacting protein pairs that share the same biological process functions and evaluated the distribution of such pairs in each network into the same pool of biological process annotations. Fisher's exact tests identified a larger portion of highly enriched functions with generally more significant p-values than the results from single-node/protein function analysis in each of the stratified subnetworks compared with interacting protein pairs in the total network [**Supp. Figure 2 in **Additional file 1]. While *photosynthesis *in leaves, *protein transport *in roots, and *response to heat *in seeds are still highly enriched, *protein folding*, *protein catabolic process*, as well as *ubiquitin-dependent protein catabolic process *become universally and significantly enriched in all five tissues of the plant. The enriched functions in each of the tissue-specific subnetworks are consistent with previous studies [36].

We further investigated general differences in protein functional enrichment among the total network and stratified subnetworks, using GO slim high-level annotations by TAIR and TIGR [42,47] [**Table S8 in **Additional file 2]. If proteins in the total network differ in functional enrichment from those of level-2 coarse-stratified organs and cell culture subnetworks and the five level-3 stratified tissue-specific subnetworks, we should expect a difference in among-functional category distributions between GO functional annotations from each of the stratified subnetworks and those from the total network. χ^2 ^tests showed such among-biological processes GO term distributions are significantly different (p-value < 1e-5) between protein annotation counts from the unstratified total network and each of the stratified subnetworks [Figure 6]. Using interacting pair annotation counts, χ^2 ^tests identified similar yet more statistically significant results for comparing the distributions of protein interacting pair annotations among networks [**Supp. Figure 2 in **Additional file 1]. In addition, a different among-network distribution between proteins of a specific GO term and the total number of annotated proteins is observable. For instance, protein annotation counts in all 15 GO slim terms of Molecular Function category form a distribution among 18 networks. Annotation counts in GO slim term *nucleotide binding *of Molecular Function form another distribution among networks, which is significantly different from the distribution of all Molecular Function protein annotations, with a p-value of 2.0e-8 by a χ^2 ^test. Similar tests show that the majority of GO terms in biological process, molecular function, and cellular component all have those significantly different distributions [Figure 6]. Using interacting pair annotation counts, instead of single protein annotation counts, an even larger portion of GO terms shows significantly different distributions [**Supp. Figure 2 in **Additional file 1].

These results suggest that over-represented protein functions in the total network are often different from those in subnetworks; therefore, the enriched functions of a conglomerate network do not reflect the true functions of context-dependent networks.

### The Total Network and Stratified Subnetworks Differ in Modular Structures

Modules, or communities in molecular networks, are expected to correspond to functional units [1,48-53], which are commonly analyzed. To extract modules from networks, we used three different clustering algorithms to partition each of the 18 networks in the 4-level hierarchy: a simulated annealing-based algorithm to optimize a defined modularity score [49]; a density-based network clustering algorithm [48], which identifies clique-like components (densely connected subnetworks) in a network as modules; an edge betweenness-based partitioning method [50] partitioning a network into modular structures by iteratively removing interactions of the highest betweenness scores.

**Figure 7:**
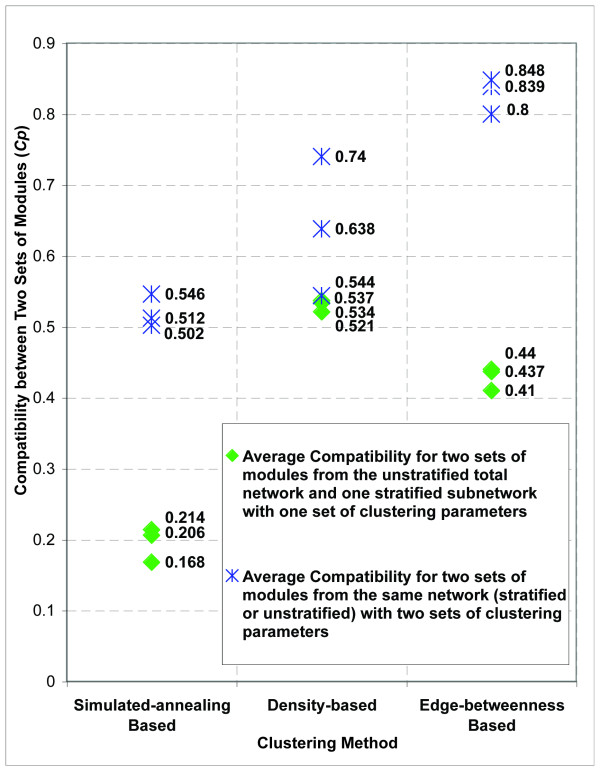
**Modules from the unstratified total network and stratified subnetworks show low compatibility**. Using each of the three clustering algorithms, modules were extracted from the unstratified total network, coarse-stratified cell culture subnetwork, and moderate-stratified tissue-specific subnetworks. Modular Compatibility scores *Cp *were calculated between two sets of modules. Green dots are average *Cp *calculated between modules extracted from the total network and those from each of the stratified subnetworks, with the same parameter settings applied for clustering. Blue stars are average *Cp *between two sets of modules from the same network (the total network, or any subnetwork) with different parameter settings for clustering. Because the density-based clustering method allows overlaps among modules, i.e., proteins or interactions may belong to multiple modules, and thus counted multiple times, which leads to higher overlaps and *Cp *scores between modules. Therefore, the separation of corresponding average *Cp *scores is not as explicit as those of results based on the other two clustering methods.

If network topology changes between the total network and stratified subnetworks, we should expect modules extracted from the total network to be different from those extracted from any of the stratified subnetworks. In order to measure the network modular compatibility, we defined a Modular Compatibility score, *Cp*, as an indication of agreement or overlap between two sets of modules (See Methods). *Cp *ranges from 0 to 1. The larger the Compatibility score is, the higher degree of overlap exists between two sets of modules. Using each of the three clustering methods, we found that modules from the total network and modules from any of the stratified subnetworks (a level-3 tissue-specific network, or the level-2 cell culture network) are much less compatible compared with modules from the same network with different sets of parameters for clustering (p-value < 1e-2 using Mann-Whitney U test) [Figure 7]. Using clustering results from the simulated annealing-based method [49] as an example, the compatibility score between a stratified tissue-specific subnetwork and the total network is 0.19, on average. In contrast, average compatibility scores are much higher for two sets of modules from the same network among seven networks (the total network, the coarse-stratified cell culture network, and the five stratified tissue-specific subnetworks) by using different sets of clustering parameters: 0.51, 0.55 and 0.50 for three comparisons respectively (p = 3.2e-11 using Mann-Whitney U test) [**Table S9 in **Additional file 2]. The low modular compatibility between the total network and stratified subnetworks suggest that modules in conglomerate networks may be largely different from those in context-dependent networks.

### The Total Network and Stratified Subnetworks Differ in Modular Functional Enrichment

We evaluated the GO function distribution of single proteins as well as protein interacting pairs in modules extracted from each network, based on the same biological process annotation terms as those used for functional enrichment analysis in stratified subnetworks. Using modules extracted by the simulated-annealing based method as an example, Fisher's exact tests show that, in general, proteins in the majority of modules clustered from the total network and the five level-3 stratified tissue-specific subnetworks are enriched in at least one GO biological process term. The percentages of modules enriched for the total network, and roots, leaves, flowers, siliques and seeds subnetworks are 83.6% (51 out of 61 modules), 90.9% (30 out of 33), 100% (33 out of 33), 85.1% (40 out of 47), 86.4% (38 out of 44), and 96.6% (28 out of 29), respectively. Considering the heterogeneity of components in modules extracted from the total network, i.e., proteins and interactions in the total network are combined from those under different contexts, it is reasonable that the percentage of functionally enriched modules is the lowest for the total network. Twenty-six biological process functional terms are universally enriched by proteins in modules from all six networks (the total network and five tissue-specific subnetworks), such as *glycolysis*, *translation*, *transport*, and *protein folding*; whereas, modules extracted from each of the five tissue-specific subnetworks have a number of exclusively enriched GO terms, representing tissue-specific functions, for example *proton transport *in roots, *photosynthetic electron transport chain *in leaves, *negative regulation of flower development *in flowers, *phospholipid biosynthetic process *in siliques, and *anaerobic respiration *in seeds [**Table S10A in **Additional file 2 and 3]. In addition, a number of modules from the total network are observed to have enriched GO terms that are not over-represented in any module from any tissue-specific subnetwork [**Table S10A in **Additional file 2 and 3]. This suggests that such modules and corresponding enriched functions may be artifacts due to the assembly of conglomerate networks by combining PPIs from various contexts, as illustrated in [Figure 1]. The results are consistent when using protein interacting pair annotations for modular functional enrichment [**Table S10B in **Additional file 2 and 3]. Similar results were obtained when evaluating modular functional enrichment by using GO slim high-level terms [**Table S8 in **Additional file 2] of all molecular function, biological process and cellular component categories [**Table S11 in **Additional file 2].

In summary, with respect to modular structures and modular functional enrichment, results of modular analysis based on conglomerate networks are mostly not transferable to context-dependent networks.

### Effects of False Interactions

It is widely suspected that the PPIs determined from high-throughput experiments may contain a large number of false interactions, or false positives [54,55]. In a recent publication, Huang et al. estimated the false discovery rates to be up to 20% for large-scale PPI screens [56]. In another study, Wuchty also used 20% as the false interaction rate in his simulation [57]. Therefore, we expect a similar percentage of false PPIs to exist in our assembled conglomerate network. To address the issue of the possible influence of these false interactions in the assembled conglomerate network on the result of our analyses, we randomly replaced 10 to 20% of PPIs from the total network and performed network stratification and the same comparative analyses on networks with replaced PPIs. The results are generally consistent with those from network analysis without replaced PPIs. For example, the percentages of network hub status change with 10% replaced interactions are highly similar to those of original networks, for both "hubs to non-hubs" and "non-hubs to hubs" changes (see Additional file 4). When replaced interactions were increased to 20%, a slight increase about 3% in "non-hubs to hubs" change and a slight decrease about 5% in "hubs to non-hubs" change are observable [**Supp. Figure 3 in **Additional file 1]. When replaced interactions were further increased to 30 or 40%, although characteristics of modified networks become deviated from the original ones, for example the clustering coefficient drops significantly from ~0.3 to 0.06 when 40% interactions were replaced in the modified networks, the analysis results are still generally consistent while being different from random expectations (Additional file 4). These results indicate the robustness of our analyses against false PPIs.

On the other hand, currently identified PPIs may be only a portion of the entire interactome with a large number of PPIs yet to be discovered. The effects of these unidentified PPIs, or false negatives, are difficult to assess directly by simulation. In this study, in order to limit the effects of false negatives, we have incorporated as many PPIs as available into the conglomerate PPI network (including validated, predicted, and curated interactions) to achieve high data coverage. We expect that the network structure and properties of the conglomerate network provide good approximations for those of the entire interactome. Therefore, the observations made in this study will likely be valid for the entire interactome. However, this can only be verified when more true PPIs become available in the future.

### Stratification Using Human PPI Networks

We also investigated whether the conclusions obtained from *A. thaliana *PPI networks can be applicable in other organisms. Therefore, we also conducted network stratification and analyses on human PPI networks. Due to the incompleteness and heterogeneity of context-specific proteomics data in human, we limited our preliminary study in human to stratifying conglomerate human PPI networks into two subnetworks of human brain and kidney. We applied network stratification using human brain and kidney proteomics data, and comparatively analyzed various aspects among stratified brain- and kidney-specific subnetworks and static conglomerate human PPI networks. Similar approaches and methods were adopted as those applied in *A. thaliana *network analyses, and results showed that most of our conclusions from *A. thaliana *network analyses are also valid in human PPI networks. For example, network hub and bottleneck status changes during stratification in human networks are similar to those in *A. thaliana *networks [**Supp. Figure 4 in **Additional file 1]. Generally, stratified brain and kidney subnetworks are different from conglomerate networks in network statistical characteristics, topological properties, including hub/bottleneck status changes, and modular structures. Detailed data can be found in Additional file 5.

## Discussion

The main reason we postulate that conglomerate networks do not reflect the true structural and functional properties of context-dependent networks is that context-dependent and meaningful biological information may be lost or misinterpreted in conglomerate networks. As implied by our results, when analyzing molecular networks, for example PPI networks, for studies focusing on specific conditions or tissues, using conglomerate networks for analysis may create errors. Ideally, rather than analyzing a conglomerate network, using stratified context-specific networks, meaningful information can be truly retrieved. On the other hand, over-stratification, i.e., network stratification based on a limited amount of highly context-specific datasets, should be avoided, because the incompleteness of context-specific information may lead to additional errors in the system-level view of the corresponding dynamics.

It is very important to note that our results, however, by no means suggest that the results from previous works on analyzing conglomerate networks are necessarily incorrect. First, the validity of studies depends on which aspect is analyzed [Table 1]. For example, for studies focusing on the identification of network hubs/bottlenecks, the results based on conglomerate networks are generally correct because the majority of network hubs/bottlenecks still remain as hubs/bottlenecks in the subnetworks. Second, to take into account the dynamic nature of context-dependent networks, many researchers have integrated other genomic datasets to assist network analysis for the retrieval of context-dependent information. As an alternative to incorporate proteomics data, researchers may combine other types of molecular data, for example microarray gene expression data, with a conglomerate network to approximate true dynamics of biological processes with reduced errors. This approach is currently successful. For instance, Calvano et al. incorporated temporal changes of gene expression related to responses to endotoxin infusion into a curated human PPI network, and better elucidated underlying mechanisms of human systemic inflammation [58]. Integrating breast cancer gene expression with human PPI networks, two groups identified novel prognostic subnetwork biomarkers for breast cancer metastasis with improved predictive power [18,59].

**Table 1:** Summary: the validity of current network analysis on static conglomerate networks

Network Analysis		Valid?	How different are the results?
**Topological Characteristics**	Degree	No	Network statistics except average clustering coefficient are generally significantly different between the total network and stratified subnetworks
		
	Betweenness	No	
		
	Clustering Coefficient	To some degree	
		
	Eccentricity	No	

**Functional Enrichment in Network**		No	Proteins in different networks are enriched in context-specific functions

**Network Hubs**	Hubs in Conglomerate Networks	To some degree	16% hubs in the total network change into non-hubs in subnetworks on average
	
	Non-hubs in Conglomerate Networks	Yes	0.8% hubs in subnetworks are non-hubs in the total network on average, which correspond to a trivial portion (< 0.1%) of non-hubs in the total network

**Network Bottlenecks**	Bottlenecks in Conglomerate Networks	To some degree	13% bottlenecks in the total network change into non-bottlenecks in subnetworks on average
	
	Non-bottlenecks in Conglomerate Networks	Yes	9% bottlenecks in subnetworks are non-bottlenecks in the total network on average, which correspond to a trivial portion of non-bottlenecks in the total network

**Modular Compatibility**		No	Modules from the total network and those from subnetworks have low Modular Compatibility

**Functional Enrichment in Modules**		No	Modules from different networks differ in over-represented context-specific functions

Network stratification may help resolve many controversies in current network biology. For instance, as implied from our results, unreasonably big hubs (nodes that have a huge number of interacting partners in a network) in conglomerate networks [23] may be a result of combining interactions under different conditions, under which these hubs may have only a moderately large number of interactions. Another example is that, contrary to the common belief that network modules should correspond to functional units, structural modules extracted from yeast PPI networks showed lack of functional enrichment [60]. This controversy may be resolved if the study were performed on condition-specific stratified subnetworks, because based on our results, modules extracted from subnetworks have higher functional enrichment than those from the static network.

Recently, there are debates over whether network hubs fall into two distinct categories of different topological characteristics, specifically named "party" or "date" hubs, as defined by having simultaneous or asynchronous interactions, respectively [32,59,61-63]. From the stratification perspective, our results suggest that the hub status of individual proteins may change among networks corresponding to diverse contexts, specific or combinatorial, and therefore different types of hubs may exist, only when taking into account context-dependent subnetworks but not static conglomerate networks alone even with reliable interactions.

Recent studies on revisiting false positive rates in large-scale experimental datasets [54,56] suggest false positive rates in large-scale data may not be as high as initially assessed [55]. This may be explained from the stratification perspective. A large fraction of interactions in a conglomerate network may no longer exist when they are validated under a specific experimental condition under which such interactions do not take place. These interactions could be taken as false positives; however, when considering other contexts, they may well be true interactions.

A recent study has shown that curated PPI data might not be suitable for topological analysis due to their potential sociological biases [54]. We have included these curated interactions because these interactions are commonly incorporated in conglomerate networks [18,35]. In this study, although a small portion of curated PPI data are included in the conglomerate network (1,177 vs. 42,131, or 0.028%), we expect their influence to be limited due to the small portion.

Finally, we admit that the most rigorous way to draw conclusions on the differences among networks is to compare a conglomerate network versus PPI networks determined by using the same experimental approach under each specific condition. However, such data are currently unavailable and we do not expect such data to become available soon due to the cost and expense to carry out a large-scale screen of PPIs. Our network stratification approach has provided a close approximation of the subnetworks under specific conditions and thus helps reveal useful insights on the structural and functional differences between a conglomerate network and the context-dependent networks.

## Conclusion

Using PPI networks and proteomics data in *A. thaliana*, we have carried out a proof-of-principle analysis on the structural and functional differences between a conglomerate network and the context-specific networks. We stratified a conglomerate *A. thaliana *PPI network into context-dependent networks with genome-scale tissue- and condition-specific proteomics data, and systematically analyzed topological, functional and modular differences among the conglomerate and context-dependent networks. We found that the results based on the conglomerate PPI network are often significantly different from the context-dependent subnetworks corresponding to specific tissues or conditions with respect to topological statistics, functional enrichment, and modular components. This conclusion is not particularly dependent on relatively arbitrary cutoffs (such as those defining network hubs or bottlenecks), nor on different module extraction algorithms, nor on the possible high false positive rates in the PPI networks. The consistency in our results suggests the robustness of our analyses.

Due to the limited data availability, we have also performed preliminary analysis on human PPI networks. We found that most of our conclusions are likely true in human as well. We speculate that the observed differences are also likely to exist in other molecular networks, such as gene regulatory networks. In directed networks, we also expect significant changes in network motifs between conglomerate and context-dependent networks. Our results may have implications in various other types of networks such as social networks, collaboration networks, and World Wide Web. Each of these networks could possibly be stratified into subnetworks in the dimension of time, location, or condition while context-dependent information is supplied.

## Methods

### Data Sources for Assembling the Conglomerate A. thaliana PPI Network

To assemble a conglomerate PPI network of *A. thaliana*, we combined three PPI datasets: predicted PPIs from TAIR containing 3,617 proteins and 19,979 interactions [40], curated PPIs from TAIR consisting of 770 proteins and 1,177 interactions [40], and predicted PPIs from AtPID containing 11,706 proteins and 24,418 interactions [41]. The assembled conglomerate network built has a total of 13,136 proteins and 42,131 interactions. We selected the part of the assembled conglomerate network that contains all proteins included in the proteomics data as the total network with 6,606 proteins and 22,165 interactions.

### Context-Dependent A. thaliana Proteomics Data

The large-scale *A. thaliana *proteomic dataset from Baerenfaller et al. includes 18 protein lists with respect to specific or combinatorial contexts, which form a 4-level hierarchy [36]. The top level protein list is the total protein list containing 13,029 proteins identified in any context, which corresponds to the collective of diverse temporal-spatial contexts. The two second level protein lists consist of a list of 10,902 proteins in any specific tissue/organ of *A. thaliana*, and a list of 8,698 proteins in any of the three cell culture conditions (dark, light, and light small). The third level protein lists include either tissue-specific proteins in the root, leaf, flower, silique and seed of the plant (6,125, 4,853, 9,075, 5,779 and 3,789 proteins, respectively), or condition-specific proteins that express under one of the three cell culture conditions (6,547, 6,474 and 4,472 proteins for dark, light and light small cell culture conditions, respectively). The fourth level protein lists contain sub-tissue-specific proteins, for example, 5,159 proteins that express in the root on the tenth day, or 5,215 proteins identified in the open flowers.

### Network Stratification Based on the Conglomerate A. thaliana PPI Network and Context-Dependent Proteomics Data

Generally, network stratification can be performed by first overlaying context-dependent data onto a conglomerate network, followed by selecting and combining interactions that exist in the same contexts to build context-dependent subnetworks. Here we constructed 17 *A. thaliana *PPI subnetworks corresponding to specific or combinatorial contexts [Figure 2], by overlaying large-scale proteomic lists described in Baerenfaller et al. [36] onto the assembled conglomerate PPI network, and selecting interacting proteins that both exist under certain conditions. Selected interactions under the same conditions are then combined to form subnetworks corresponding to specific contexts. The coverage of proteins in each of the networks built ranges from 43.5% (1,596 out of 3,665 proteins for the fine-stratified cotyledons subnetwork) to 53.5% (2,390 out of 4,466 proteins for the fine-stratified 23-day roots subnetwork) compared with source protein lists. For the total network, 50.7% (6,606 out of 13,029) of proteins from the source protein list are covered in the network. In this study, network stratification is performed in combined dimensions of time (proteins from different tissues are sampled at different time), location (different tissues/organs of the plant) and condition (different cell culture conditions).

### Data Sources for Human PPI Network Stratification

For the stratification of human PPI networks, we used two candidate conglomerate networks: all non-redundant PPIs from the seventh version of the HPRD database [64], including 9,305 proteins and 35,021 interactions, as well as a pooled dataset of 11,203 proteins and 57,235 interactions assembled in Chuang et al. [18], which combines PPIs from yeast two-hybrid experiments [16,65], computational predictions via orthology and co-citation [64], and curation of the literature [66-68]. Human proteomics data we used are human brain and kidney proteomic datasets from HUPO projects [69,70]. The brain proteomics data include 1,832 proteins with gene symbols that express in human brain, of which 803 proteins and 1,616 interactions, and 937 proteins and 2,674 interactions can be mapped onto the HPRD PPI network and the pooled PPI network, respectively. The kidney protein list consists of 2,950 non-redundant proteins with gene symbols, of which 1,417 proteins and 3,117 interactions, and 1,593 proteins and 6,498 interactions were present and selected from the two conglomerate networks, respectively.

### Identification of Network Hubs and Bottlenecks

In general, network hubs and bottlenecks are nodes with relatively high degree and betweenness values, respectively. Node degree is the number of edges connecting to the node; node betweenness is measured by the number of shortest paths between any pair of nodes in the network that go through the node. In this study, we quantitatively defined nodes with the top 20% of degree values as network hubs, and thus the remaining 80% of nodes are considered non-hubs. Results showed consistency in topology analysis when changing the cutoff percentage to 5 or 10% for network hub definition. For network bottlenecks, we also defined the top 5, 10 or 20% of nodes with highest betweenness values as bottlenecks, and the analysis results showed consistency. We used network tool tYNA to calculate node degree and betweenness values in each network (http://tyna.gersteinlab.org/tyna/; [71]).

### Node Degree/Betweenness Class Assignment

To measure node degree or betweenness value change among networks, we used top 5, 10, 20, 50 percent degree or betweenness values as cutoffs, and put each protein from each of the networks into one of the five classes, representing nodes of similar degree or betweenness values, respectively. To address the issue of rather arbitrariness of such classification, we also used top 10, 20, 50 as percentage cutoffs for four classes, or top 5, 15, 25, 60 as percentage cutoffs for five classes, for node degree and betweenness values, respectively. The analysis results showed consistency, which means node degree/betweenness variations for same proteins during network stratification generally exist.

### Compatibility Measurement between Two Subnetworks

We used Jaccard similarity coefficient, or Jaccard Index, to measure the compatibility, or the agreement of nodes and interactions between pairs of networks, which is defined as:

$$J_{v}=\frac{\left|A_{v}\cap B_{v}\right|}{\left|A_{v}\cup B_{v}\right|},\text{ or }J_{e}=\frac{\left|A_{e}\cap B_{e}\right|}{\left|A_{e}\cup B_{e}\right|},$$

where *A*_ν_, *B*_ν _are node sets, and *A*_*e*_, *B*_*e *_are edge sets of two networks, respectively. This index quantitatively measures the overlap of nodes or edges between two networks.

### Module Identification through Network Clustering

Modules in networks are generally defined as community-like subnetwork structures that are densely intra-connected and loosely inter-connected. Modules in molecular networks are expected to correspond to functional units [18]. Three different clustering algorithms were used to identify modules from the unstratified and stratified networks: 1) a density-based method from Palla et al., which seeks to extract all subnetworks that contain *k-cliques *(i.e., fully connected complete graphs of *k *nodes) given an integer *k *[48]; 2) a simulated annealing-based method to maximize Modularity *M*, defined as

$$M=\sum_{s=1}^{N}\left[\frac{l_{s}}{L}-{\left(\frac{k_{s}}{2L}\right)}^{2}\right],$$

where *N *is the number of modules, *L *is the total number of edges in the network, *l*_*s *_is the number of edges within module *s*, and *k*_*s *_is the sum of the degrees of the nodes in modules [49,72]. Modularity by definition seeks to maximize intra-connections and minimize inter-connections of modules in the network. This algorithm uses simulated annealing strategy to optimize the Modularity score. 3) An edge betweenness centrality-based algorithm that partitions the network iteratively to extract modules [50]. Based on the fact that edges of high betweenness centrality are often connectors between communities, this algorithm iteratively removes an edge with the highest betweenness centrality in the network. When a cutoff is selected, for example, 40 percent of edge removal, the rest of the network, usually partitioned as subnetworks, contains the resulting modules.

### Compatibility Measurement between Two Sets of Modules Extracted from Networks

Jaccard Index is not appropriate to measure modular compatibility without extension, because compatible modules from two sets of modules, i.e., modules with the largest number of node or edge overlaps, are often not symmetric. Therefore we extended Jaccard Index, and defined Compatibility Score between two sets of modules as

$$Cp=\frac{1}{2}[\sum_{i=1}^{M}\frac{C1_{i}}{T1_{i}+P1_{i}-C1_{i}}+\sum_{j=1}^{N}\frac{C2_{j}}{T2_{j}+P2_{j}-C2_{j}}],$$

where *M *and *N *are numbers of modules extracted from two networks, *i *and *j *denote module index, *T1*_*i *_(or *T2*_*j*_) is the number of nodes in each module from one of the two networks, *P1*_*i *_(or *P2*_*j*_) is the number of nodes in the most compatible module of *T1*_*i *_(or *T2*_*j*_) from the other network, and *C1*_*i *_(or *C2*_*j*_) is the number of overlapping nodes between *T1*_*i *_and *P1*_*i *_(or between *T2*_*j *_and *P2*_*j*_). Given a module A from a network, we restrict that the most compatible module B for A from another set of modules should have at least 50% of nodes in module A. In this study, a small number of modules are perfectly conserved while stratified, i.e., two modules extracted from two networks (or from one network with different clustering parameters) are identical. In addition, a large number of modules have compatible modules.

### Randomization in Network Compatibility Analysis

When measuring network compatibility, we also calculated Jaccard Indices between randomized network pairs for comparison purpose. Randomized networks were built by randomly selecting from the total network the same number of nodes or edges as those of real networks. 500 randomized networks were built for statistical tests and p-value calculations.

### Randomization in Network Statistics and Hub/Bottleneck Status Change Analysis

In order to evaluate whether the difference in network statistics, for example average degree or clustering coefficient, between stratified subnetworks and the total network, and whether "hub to non-hub", "non-hub to hub", or "bottleneck to non-bottleneck", "non-bottleneck to bottleneck" changes depend on the change of network sizes, we built randomized networks, assessed the number and percentage of hub/bottleneck status changes from the total network, and calculated p-values by Welch's t-tests and Student's t-tests for statistics and hub status changes, respectively. Twenty randomized networks were constructed by randomly selecting a similar number of proteins as in each of the tissue-specific protein lists, overlaying these random proteins onto the total network, and selecting interacting protein pairs that both exist. (In order to approximate both the node and edge numbers of the original networks, the numbers of nodes and edges in the random networks are often not identical to those in the original networks). The total degree of each of the randomized network must be within 10% of the original network. These randomized networks are scale-free, and comparable in total nodes and interactions to the original tissue-specific subnetworks.

### Replacing Interactions in Networks

In order to address the issue about the possible influence of false interactions in the assembled conglomerate *A. thaliana *PPI network. We randomly removed 10 to 40% of interactions in the unstratified total network, and replaced them with the same number of interactions between random proteins that do not exist in the unstratified network, so that the resulting networks contain 10 to 40% of replaced interactions while maintaining the same size as the total network. This approach is similar to the method used in a former study [57].

## List of abbreviations

PPI: protein-protein interaction.

## Authors' contributions

MZ designed and performed analyses, prepared the figures, and wrote the manuscript. LJL designed analyses, wrote the manuscript, and supervised the project. Both authors read and approved the final manuscript.

## Supplementary Material

Additional file 1 **Four supplementary figures**. Supp. Figure 1 shows a 4-level hierarchy of the unstratified and stratified networks. Supp. Figure 2 shows the functional enrichment for protein pairs in each of the networks. Supp. Figure 3 depicts hub status change with 20% replaced interactions. And Supp. Figure 4 shows hub/bottleneck status change in human networks. Click here for file

Additional file 2 **Eleven supplementary tables**. Detailed results from topological, functional and modular analyses are summarized in these supplementary tables. Click here for file

Additional file 3 **Universally and exclusively enriched functions in modules extracted from each of the networks**. 26 biological process functions are universally enriched in modules from the total network and those from each of the stratified subnetworks, while a number of exclusively enriched functions exist in modules extracted from each of the networks. Click here for file

Additional file 4 **Results of analyses using *A. thaliana *networks with replaced interactions**. Degree distributions were drawn in figures for each of the networks with replaced interactions. Network statistics including degree, clustering coefficient, eccentricity and betweenness were calculated and compared. Network hub/bottleneck status changes were investigated. The results are generally consistent with those based on original networks. Click here for file

Additional file 5 **Results of analyses using human PPI networks**. When using human PPI networks for analysis, results showed general consistency with the results based on *A. thaliana *networks. Network statistics including degree, clustering coefficient, eccentricity and betweenness were calculated and compared. Network compatibility was assessed. Network hub/bottleneck status changes were also investigated. Click here for file
