# Supplementary material for: Investigating the validity of current network analysis on static conglomerate networks by protein network stratification
Source: BMC Bioinformatics. 2010 Sep 16;11:466. doi: 10.1186/1471-2105-11-466 (PMC2949894; doi:10.1186/1471-2105-11-466)
Supplement: Additional file 1 — Four supplementary figures. Supp. Figure 1 shows a 4-level hierarchy of the unstratified and stratified networks. Supp. Figure 2 shows the functional enrichment for protein pairs in each of the networks. Supp. Figure 3 depicts hub status change with 20% replaced interactions. And Supp. Figure 4 shows hub/bottleneck status change in human networks. [file 1471-2105-11-466-S1.DOC]

**Supp. Fig. 1. A 4-level Hierarchy for 18 *A. thaliana* Networks during Stratification**

**
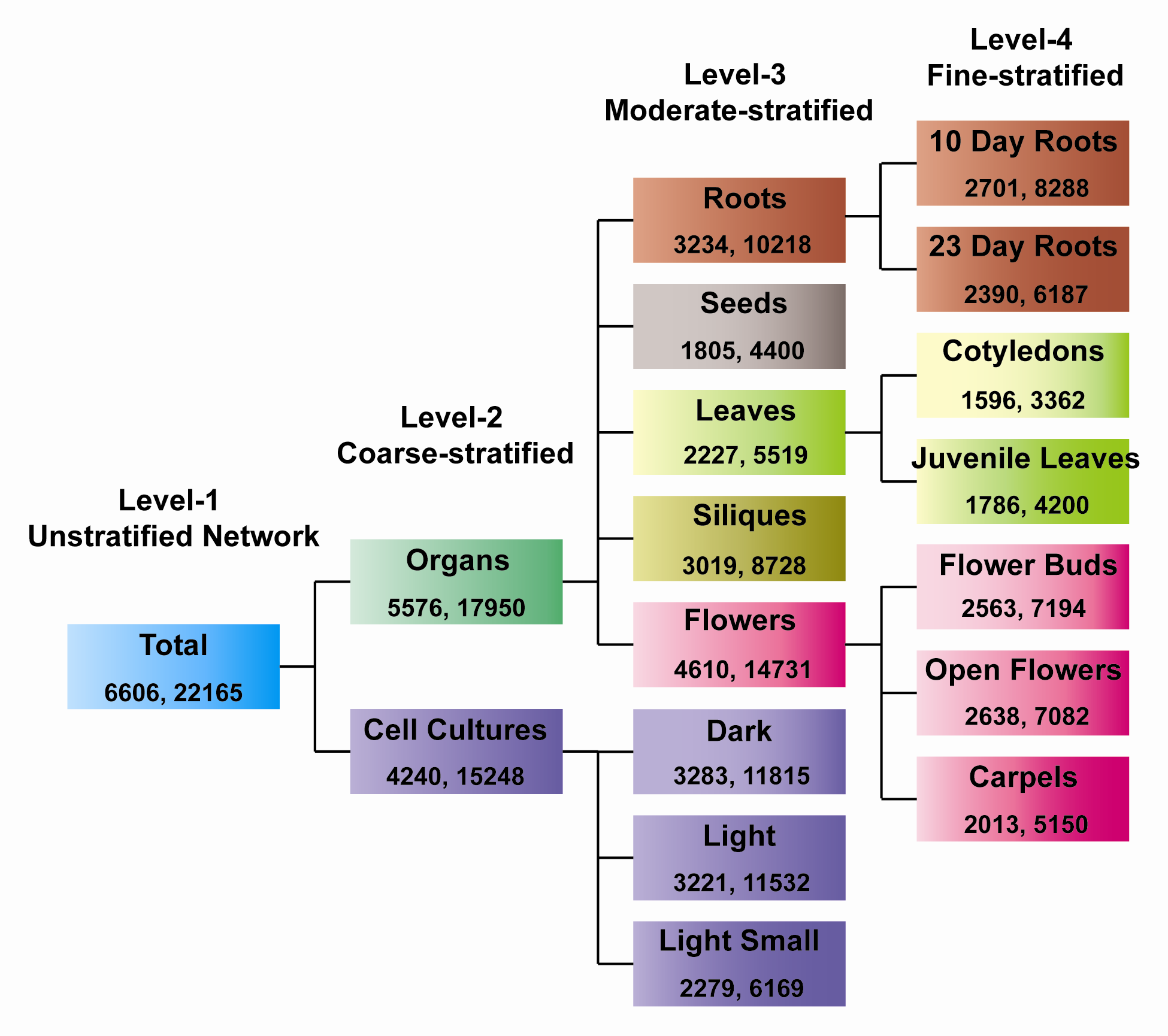
**

**Supp. Fig. 1.** Eighteen *A. thaliana* PPI networks of specific or combinatorial temporal and spatial conditions form a 4-level hierarchy, with differences in granularity during stratification. Each network contains PPIs with both proteins exist in the corresponding conditions as described in a proteomics study [1]. The two numbers in the box separated by a comma denote the number of proteins and PPIs in each network, respectively. Roots, seeds, leaves, siliques, and flowers subnetworks denote PPI networks corresponding to different tissues of the plant. “Dark”, “light” and “light small” subnetworks represent PPI networks under different cell culture conditions.

**Supp. Fig. 2. Functional Enrichment in Networks Using GO Annotations for Protein Interacting Pairs**

**
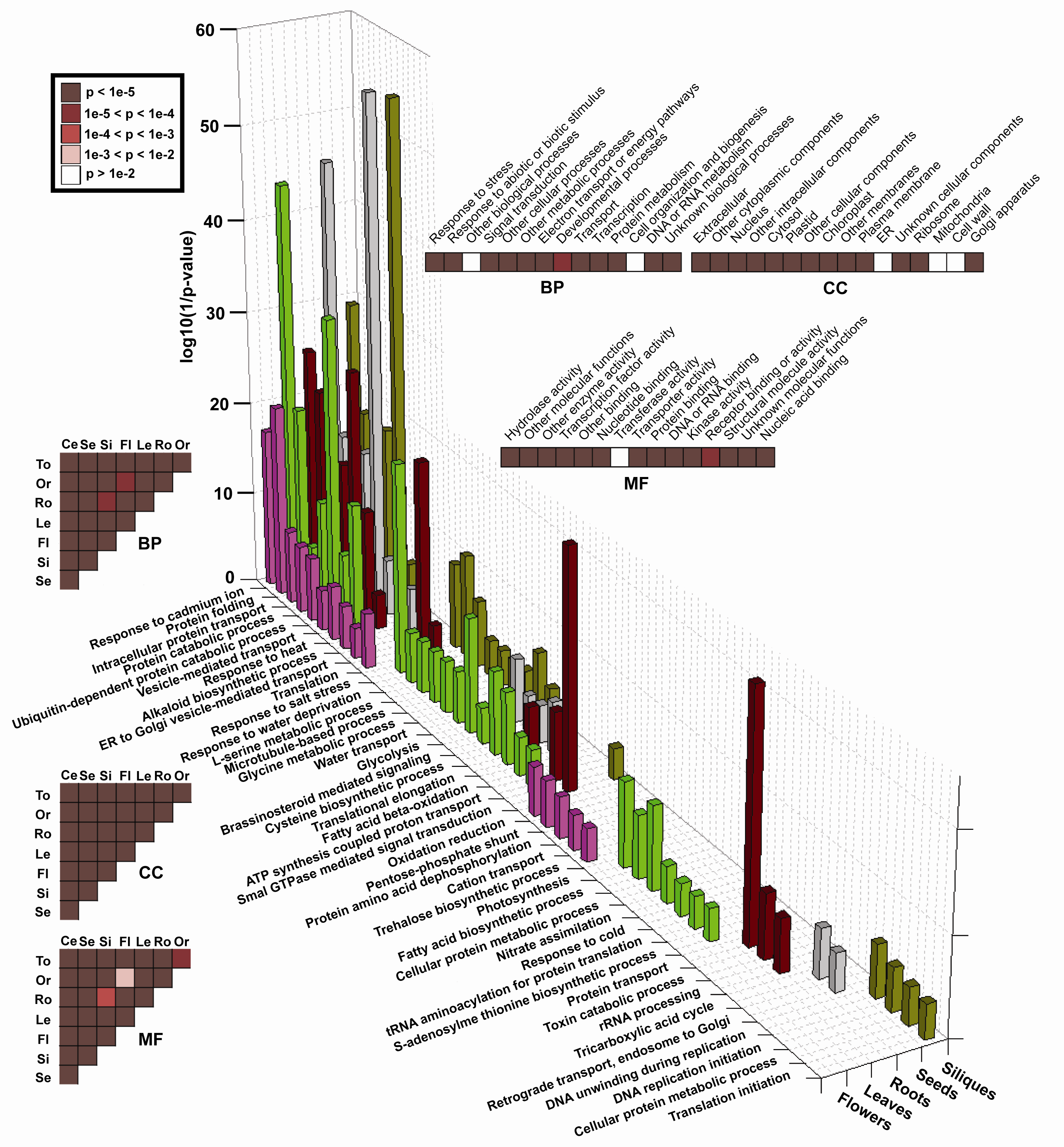
**

**Supp. Fig. 2.** Functional classification of protein interacting pairs is evaluated using the same set of GO biological process terms. Fisher’s exact tests were used to assess highly enriched GO functions. Shown are functions with p-value < 5e-4 in each of the stratified tissue-specific subnetworks. (Left) Different colors indicate significance of pairwise functional enrichment differences between protein interacting pairs in the two networks/subnetworks, using GO slim high level terms. (Top right) Different colors indicate significance of differences in the distribution of each GO slim function term by protein interacting pair annotations among networks.

**Supp. Fig. 3. Hub Status Change with 20% Replaced Interactions**

**
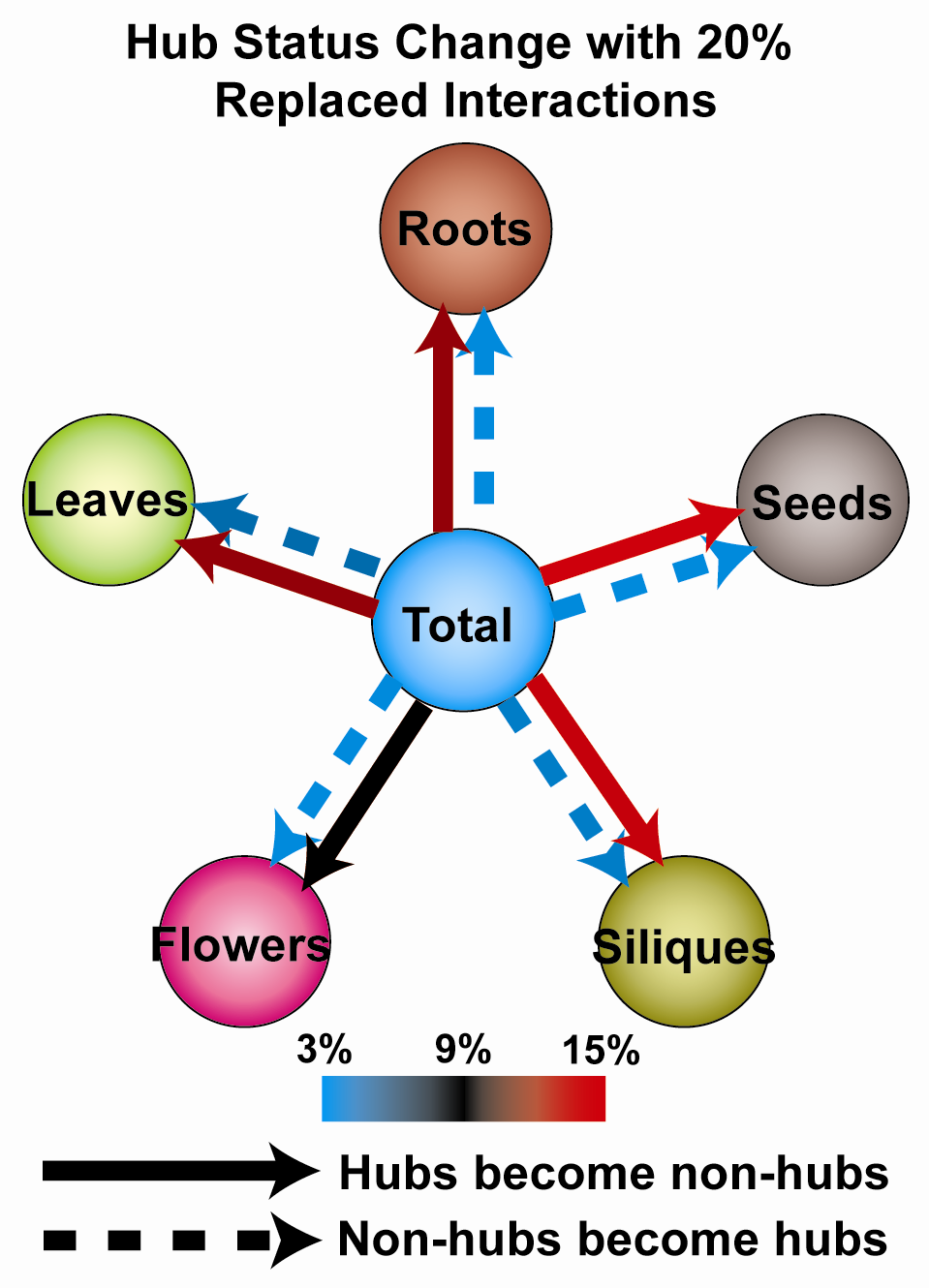
**

**Supp. Fig. 3.** Here 20% interactions in the total network are replaced with interactions between random proteins, and subnetworks are stratified from this total network. The percentages of hub status change are similar to those based on original networks. Yet, both a slight increase in “non-hubs to hubs” change and a slight decrease in “hubs to non-hubs” change exist. Results based on networks with 10% replaced interactions are highly similar to those of original networks (see **Additional file 4**).

**Supp.** **Fig. 4. Hub/Bottleneck Status Change in Human Networks**

**
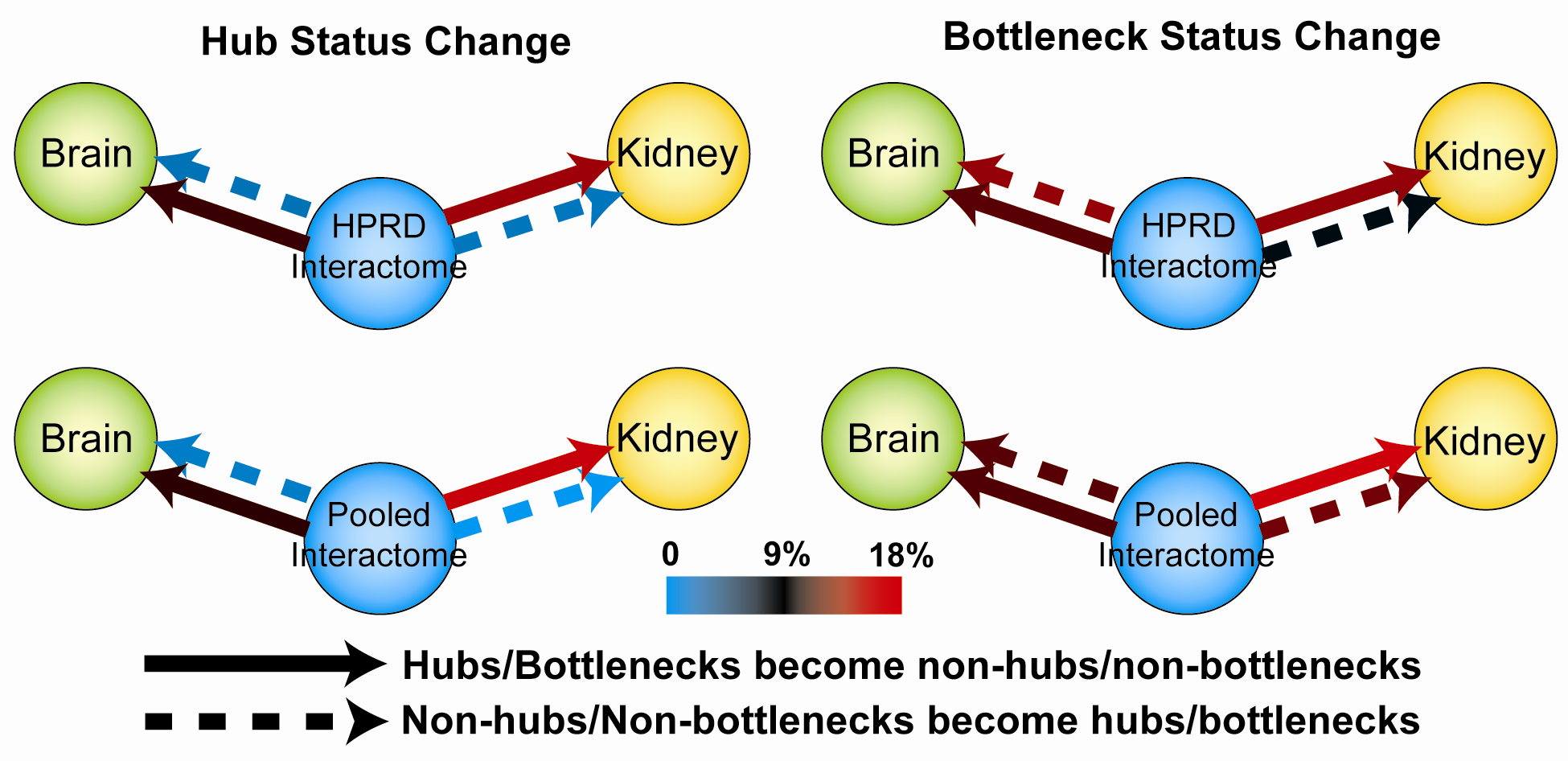
**

**Supp.** **Fig. 4.** Each ball is a human PPI network. Two conglomerate networks used are a human interactome based on the HPRD database (Version 7) [2], consisting of 9,305 proteins and 35,021 interactions, as well as a pooled human interactome from Chuang et al. [3], consisting of 11,203 proteins and 57,235 interactions. The percentages of “hubs to non-hubs” and “non-hubs to hubs” changes are similar to the results based on *A. thaliana* networks.

**References**

[1] Baerenfaller K, Grossmann J, Grobei MA, Hull R, Hirsch-Hoffmann M, Yalovsky S, Zimmermann P, Grossniklaus U, Gruissem W, Baginsky S: **Genome-scale proteomics reveals Arabidopsis thaliana gene models and proteome dynamics.** *Science* 2008, **320:**938-941.

[2] Ramani AK, Bunescu RC, Mooney RJ, Marcotte EM: **Consolidating the set of known human protein-protein interactions in preparation for large-scale mapping of the human interactome.** *Genome Biol* 2005, **6:**R40.

[3] Chuang HY, Lee E, Liu YT, Lee D, Ideker T: **Network-based classification of breast cancer metastasis.** *Mol Syst Biol* 2007, **3:**140.
