# Supplementary material for: Investigating the validity of current network analysis on static conglomerate networks by protein network stratification
Source: BMC Bioinformatics. 2010 Sep 16;11:466. doi: 10.1186/1471-2105-11-466 (PMC2949894; doi:10.1186/1471-2105-11-466)
Supplement: Additional file 3 — Universally and exclusively enriched functions in modules extracted from each of the networks. 26 biological process functions are universally enriched in modules from the total network and those from each of the stratified subnetworks, while a number of exclusively enriched functions exist in modules extracted from each of the networks. [file 1471-2105-11-466-S3.DOC]

**Additional file 3. Universally and Exclusively Enriched Functions in Modules from Specific Networks**

**Universally and Exclusively Enriched Functions in Modules from Specific Networks Using Annotations for Proteins**

GO BP terms that are universally enriched in the total network and five tissue-specific subnetworks:

GO:0006096 glycolysis

GO:0015031 protein transport

GO:0008152 metabolic process

GO:0009408 response to heat

GO:0006606 protein import into nucleus

GO:0006014 D-ribose metabolic process

GO:0007264 small GTPase mediated signal transduction

GO:0006810 transport

GO:0044267 cellular protein metabolic process

GO:0016192 vesicle-mediated transport

GO:0006635 fatty acid beta-oxidation

GO:0009058 biosynthetic process

GO:0006544 glycine metabolic process

GO:0006457 protein folding

GO:0006413 translational initiation

GO:0019344 cysteine biosynthetic process

GO:0006412 translation

GO:0055114 oxidation reduction

GO:0006511 ubiquitin-dependent protein catabolic process

GO:0006633 fatty acid biosynthetic process

GO:0010388 cullin deneddylation

GO:0006563 L-serine metabolic process

GO:0006886 intracellular protein transport

GO:0006508 proteolysis

GO:0009742 brassinosteroid mediated signaling

GO:0006888 ER to Golgi vesicle-mediated transport

Enriched GO BP terms in the total network but not in any subnetworks:

GO:0019760 glucosinolate metabolic process

GO:0006334 nucleosome assembly

GO:0008150 biological_process

GO:0045038 protein import into chloroplast thylakoid membrane

GO:0006790 sulfur metabolic process

GO:0006396 RNA processing

GO:0009958 positive gravitropism

GO:0006874 cellular calcium ion homeostasis

GO:0022900 electron transport chain

GO:0009638 phototropism

GO:0048437 floral organ development

GO:0006349 genetic imprinting

GO:0009736 cytokinin mediated signaling

GO:0006821 chloride transport

GO:0007031 peroxisome organization

GO:0008272 sulfate transport

GO:0019722 calcium-mediated signaling

GO:0016226 iron-sulfur cluster assembly

GO:0045449 regulation of transcription

GO:0009617 response to bacterium

GO:0000160 two-component signal transduction system (phosphorelay)

GO:0009294 DNA mediated transformation

GO:0006839 mitochondrial transport

GO:0007165 signal transduction

GO:0051726 regulation of cell cycle

GO:0010017 red or far red light signaling pathway

GO:0006298 mismatch repair

GO:0009626 plant-type hypersensitive response

GO:0010216 maintenance of DNA methylation

GO:0006352 transcription initiation

GO:0010332 response to gamma radiation

GO:0006828 manganese ion transport

GO:0010020 chloroplast fission

GO:0006617 "SRP-dependent cotranslational protein targeting to membrane, signal sequence recognition"

GO:0000724 double-strand break repair via homologous recombination

GO:0006367 transcription initiation from RNA polymerase II promoter

GO:0006355 "regulation of transcription, DNA-dependent"

GO:0009960 endosperm development

GO:0007018 microtubule-based movement

GO:0006875 cellular metal ion homeostasis

GO:0009934 regulation of meristem organization

Root-specific enriched GO BP terms in roots network:

GO:0009116 nucleoside metabolic process

GO:0010387 signalosome assembly

GO:0006519 cellular amino acid and derivative metabolic process

GO:0019430 removal of superoxide radicals

GO:0010107 potassium ion import

GO:0006749 glutathione metabolic process

GO:0009620 response to fungus

GO:0006464 protein modification process

GO:0000338 protein deneddylation

GO:0019509 methionine salvage

GO:0000910 cytokinesis

GO:0016481 negative regulation of transcription

GO:0009407 toxin catabolic process

GO:0006896 Golgi to vacuole transport

GO:0015914 phospholipid transport

GO:0015992 proton transport

GO:0006751 glutathione catabolic process

GO:0006086 acetyl-CoA biosynthetic process from pyruvate

Enriched GO BP terms in roots network but not total network:

GO:0009116 nucleoside metabolic process

GO:0007021 tubulin complex assembly

GO:0009051 "pentose-phosphate shunt, oxidative branch"

GO:0009821 alkaloid biosynthetic process

GO:0010387 signalosome assembly

GO:0006519 cellular amino acid and derivative metabolic process

GO:0006006 glucose metabolic process

GO:0006749 glutathione metabolic process

GO:0006486 protein amino acid glycosylation

GO:0009620 response to fungus

GO:0010623 developmental programmed cell death

GO:0042546 cell wall biogenesis

GO:0019509 methionine salvage

GO:0006378 mRNA polyadenylation

GO:0045454 cell redox homeostasis

GO:0051605 protein maturation by peptide bond cleavage

GO:0006090 pyruvate metabolic process

GO:0031087 deadenylation-independent decapping of nuclear-transcribed mRNA

GO:0000910 cytokinesis

GO:0016481 negative regulation of transcription

GO:0006414 translational elongation

GO:0015865 purine nucleotide transport

GO:0042819 vitamin B6 biosynthetic process

GO:0015992 proton transport

GO:0006995 cellular response to nitrogen starvation

GO:0006751 glutathione catabolic process

GO:0008295 spermidine biosynthetic process

GO:0042732 D-xylose metabolic process

GO:0006086 acetyl-CoA biosynthetic process from pyruvate

Leaf-specific enriched GO BP terms in leaves network:

GO:0006423 cysteinyl-tRNA aminoacylation

GO:0007033 vacuole organization

GO:0042325 regulation of phosphorylation

GO:0006529 asparagine biosynthetic process

GO:0006535 cysteine biosynthetic process from serine

GO:0009767 photosynthetic electron transport chain

GO:0009793 embryonic development ending in seed dormancy

GO:0010143 cutin biosynthetic process

Enriched GO BP terms in leaves network but not total network:

GO:0045037 protein import into chloroplast stroma

GO:0006423 cysteinyl-tRNA aminoacylation

GO:0009821 alkaloid biosynthetic process

GO:0031998 regulation of fatty acid beta-oxidation

GO:0009744 response to sucrose stimulus

GO:0007033 vacuole organization

GO:0015979 photosynthesis

GO:0042325 regulation of phosphorylation

GO:0000302 response to reactive oxygen species

GO:0006535 cysteine biosynthetic process from serine

GO:0009793 embryonic development ending in seed dormancy

GO:0048366 leaf development

GO:0006879 cellular iron ion homeostasis

GO:0006378 mRNA polyadenylation

GO:0045454 cell redox homeostasis

GO:0055072 iron ion homeostasis

GO:0006826 iron ion transport

GO:0006090 pyruvate metabolic process

GO:0008361 regulation of cell size

GO:0010039 response to iron ion

GO:0010540 basipetal auxin transport

GO:0019253 reductive pentose-phosphate cycle

GO:0006510 ATP-dependent proteolysis

GO:0006744 ubiquinone biosynthetic process

GO:0006414 translational elongation

GO:0015865 purine nucleotide transport

GO:0042819 vitamin B6 biosynthetic process

GO:0043481 anthocyanin accumulation in tissues in response to UV light

GO:0006995 cellular response to nitrogen starvation

GO:0008295 spermidine biosynthetic process

Flower-specific enriched GO BP terms in flowers network:

GO:0010267 "RNA interference, production of ta-siRNAs"

GO:0007067 mitosis

GO:0009910 negative regulation of flower development

GO:0006499 N-terminal protein myristoylation

GO:0030422 "RNA interference, production of siRNA"

GO:0009723 response to ethylene stimulus

GO:0006306 DNA methylation

GO:0009750 response to fructose stimulus

GO:0043087 regulation of GTPase activity

GO:0006913 nucleocytoplasmic transport

GO:0009738 abscisic acid mediated signaling

GO:0000085 G2 phase of mitotic cell cycle

GO:0006221 pyrimidine nucleotide biosynthetic process

GO:0006468 protein amino acid phosphorylation

GO:0009218 pyrimidine ribonucleotide metabolic process

GO:0009616 virus induced gene silencing

GO:0045900 negative regulation of translational elongation

GO:0006470 protein amino acid dephosphorylation

GO:0019761 glucosinolate biosynthetic process

GO:0006890 "retrograde vesicle-mediated transport, Golgi to ER"

GO:0010617 circadian regulation of calcium ion oscillation

GO:0040007 growth

Flower-specific enriched GO BP terms not in roots/leaves network:

GO:0006268 DNA unwinding during replication

GO:0010267 "RNA interference, production of ta-siRNAs"

GO:0007067 mitosis

GO:0009910 negative regulation of flower development

GO:0044237 cellular metabolic process

GO:0045941 positive regulation of transcription

GO:0006499 N-terminal protein myristoylation

GO:0030422 "RNA interference, production of siRNA"

GO:0006979 response to oxidative stress

GO:0009723 response to ethylene stimulus

GO:0009168 purine ribonucleoside monophosphate biosynthetic process

GO:0006397 mRNA processing

GO:0006306 DNA methylation

GO:0009750 response to fructose stimulus

GO:0009850 auxin metabolic process

GO:0043087 regulation of GTPase activity

GO:0006913 nucleocytoplasmic transport

GO:0009738 abscisic acid mediated signaling

GO:0006013 mannose metabolic process

GO:0000085 G2 phase of mitotic cell cycle

GO:0006221 pyrimidine nucleotide biosynthetic process

GO:0016126 sterol biosynthetic process

GO:0006468 protein amino acid phosphorylation

GO:0006270 DNA replication initiation

GO:0009081 branched chain family amino acid metabolic process

GO:0009218 pyrimidine ribonucleotide metabolic process

GO:0009067 aspartate family amino acid biosynthetic process

GO:0009616 virus induced gene silencing

GO:0006099 tricarboxylic acid cycle

GO:0008283 cell proliferation

GO:0045900 negative regulation of translational elongation

GO:0009809 lignin biosynthetic process

GO:0006470 protein amino acid dephosphorylation

GO:0009411 response to UV

GO:0019761 glucosinolate biosynthetic process

GO:0019243 methylglyoxal catabolic process to D-lactate

GO:0006890 "retrograde vesicle-mediated transport, Golgi to ER"

GO:0006446 regulation of translational initiation

GO:0010617 circadian regulation of calcium ion oscillation

GO:0040007 growth

Enriched GO BP terms in flowers network but not total network:

GO:0010267 "RNA interference, production of ta-siRNAs"

GO:0009821 alkaloid biosynthetic process

GO:0006006 glucose metabolic process

GO:0045941 positive regulation of transcription

GO:0030422 "RNA interference, production of siRNA"

GO:0006486 protein amino acid glycosylation

GO:0009723 response to ethylene stimulus

GO:0010623 developmental programmed cell death

GO:0009168 purine ribonucleoside monophosphate biosynthetic process

GO:0006306 DNA methylation

GO:0009750 response to fructose stimulus

GO:0000085 G2 phase of mitotic cell cycle

GO:0006090 pyruvate metabolic process

GO:0031087 deadenylation-independent decapping of nuclear-transcribed mRNA

GO:0008361 regulation of cell size

GO:0010540 basipetal auxin transport

GO:0009616 virus induced gene silencing

GO:0006744 ubiquinone biosynthetic process

GO:0006099 tricarboxylic acid cycle

GO:0019243 methylglyoxal catabolic process to D-lactate

GO:0043481 anthocyanin accumulation in tissues in response to UV light

GO:0040007 growth

Silique-specific enriched GO BP terms in siliques network:

GO:0008654 phospholipid biosynthetic process

GO:0006595 polyamine metabolic process

GO:0009186 deoxyribonucleoside diphosphate metabolic process

GO:0006970 response to osmotic stress

GO:0006891 intra-Golgi vesicle-mediated transport

GO:0019762 glucosinolate catabolic process

GO:0006301 postreplication repair

GO:0030149 sphingolipid catabolic process

GO:0010204 "defense response signaling pathway, resistance gene-independent"

GO:0009624 response to nematode

GO:0008652 cellular amino acid biosynthetic process

GO:0051276 chromosome organization

GO:0007049 cell cycle

Silique-specific enriched GO BP terms not in roots/leaves network:

GO:0006268 DNA unwinding during replication

GO:0008654 phospholipid biosynthetic process

GO:0044237 cellular metabolic process

GO:0006595 polyamine metabolic process

GO:0009186 deoxyribonucleoside diphosphate metabolic process

GO:0006970 response to osmotic stress

GO:0006891 intra-Golgi vesicle-mediated transport

GO:0019762 glucosinolate catabolic process

GO:0009168 purine ribonucleoside monophosphate biosynthetic process

GO:0006013 mannose metabolic process

GO:0006301 postreplication repair

GO:0030149 sphingolipid catabolic process

GO:0016126 sterol biosynthetic process

GO:0010204 "defense response signaling pathway, resistance gene-independent"

GO:0006270 DNA replication initiation

GO:0009624 response to nematode

GO:0009081 branched chain family amino acid metabolic process

GO:0008652 cellular amino acid biosynthetic process

GO:0009067 aspartate family amino acid biosynthetic process

GO:0008283 cell proliferation

GO:0009809 lignin biosynthetic process

GO:0009411 response to UV

GO:0051276 chromosome organization

GO:0019243 methylglyoxal catabolic process to D-lactate

GO:0006446 regulation of translational initiation

GO:0007049 cell cycle

Enriched GO BP terms in siliques network but not total network:

GO:0007021 tubulin complex assembly

GO:0009051 "pentose-phosphate shunt, oxidative branch"

GO:0008654 phospholipid biosynthetic process

GO:0009821 alkaloid biosynthetic process

GO:0006595 polyamine metabolic process

GO:0006006 glucose metabolic process

GO:0031998 regulation of fatty acid beta-oxidation

GO:0009744 response to sucrose stimulus

GO:0009186 deoxyribonucleoside diphosphate metabolic process

GO:0006486 protein amino acid glycosylation

GO:0006970 response to osmotic stress

GO:0019762 glucosinolate catabolic process

GO:0009168 purine ribonucleoside monophosphate biosynthetic process

GO:0042546 cell wall biogenesis

GO:0006879 cellular iron ion homeostasis

GO:0006378 mRNA polyadenylation

GO:0055072 iron ion homeostasis

GO:0006826 iron ion transport

GO:0051605 protein maturation by peptide bond cleavage

GO:0006301 postreplication repair

GO:0030149 sphingolipid catabolic process

GO:0010204 "defense response signaling pathway, resistance gene-independent"

GO:0006090 pyruvate metabolic process

GO:0031087 deadenylation-independent decapping of nuclear-transcribed mRNA

GO:0009624 response to nematode

GO:0010039 response to iron ion

GO:0019253 reductive pentose-phosphate cycle

GO:0008652 cellular amino acid biosynthetic process

GO:0006414 translational elongation

GO:0015865 purine nucleotide transport

GO:0042819 vitamin B6 biosynthetic process

GO:0019243 methylglyoxal catabolic process to D-lactate

GO:0006995 cellular response to nitrogen starvation

GO:0042732 D-xylose metabolic process

Seed-specific enriched GO BP terms in seeds network:

GO:0009061 anaerobic respiration

GO:0030036 actin cytoskeleton organization

GO:0010188 response to microbial phytotoxin

GO:0006950 response to stress

GO:0001676 long-chain fatty acid metabolic process

Seed-specific enriched GO BP terms not in roots/leaves network:

GO:0009061 anaerobic respiration

GO:0030036 actin cytoskeleton organization

GO:0045941 positive regulation of transcription

GO:0006979 response to oxidative stress

GO:0006397 mRNA processing

GO:0009850 auxin metabolic process

GO:0006013 mannose metabolic process

GO:0010188 response to microbial phytotoxin

GO:0006950 response to stress

GO:0006099 tricarboxylic acid cycle

GO:0001676 long-chain fatty acid metabolic process

GO:0006446 regulation of translational initiation

Enriched GO BP terms in seeds network but not total network:

GO:0045037 protein import into chloroplast stroma

GO:0009821 alkaloid biosynthetic process

GO:0009061 anaerobic respiration

GO:0006006 glucose metabolic process

GO:0030036 actin cytoskeleton organization

GO:0045941 positive regulation of transcription

GO:0015979 photosynthesis

GO:0000302 response to reactive oxygen species

GO:0048366 leaf development

GO:0006879 cellular iron ion homeostasis

GO:0055072 iron ion homeostasis

GO:0006826 iron ion transport

GO:0010039 response to iron ion

GO:0006510 ATP-dependent proteolysis

GO:0006950 response to stress

GO:0006414 translational elongation

GO:0015865 purine nucleotide transport

GO:0006099 tricarboxylic acid cycle

GO:0008295 spermidine biosynthetic process

**Universally and Exclusively Enriched Functions in Modules from Specific Networks Using Annotations for Protein Interacting Pairs**

GO BP terms that are universally enriched in every network:

GO:0006096 glycolysis

GO:0009821 alkaloid biosynthetic process

GO:0015031 protein transport

GO:0008152 metabolic process

GO:0009408 response to heat

GO:0006606 protein import into nucleus

GO:0009970 cellular response to sulfate starvation

GO:0007264 small GTPase mediated signal transduction

GO:0005975 carbohydrate metabolic process

GO:0006810 transport

GO:0042147 "retrograde transport, endosome to Golgi"

GO:0030163 protein catabolic process

GO:0015986 ATP synthesis coupled proton transport

GO:0044267 cellular protein metabolic process

GO:0009086 methionine biosynthetic process

GO:0006414 translational elongation

GO:0016192 vesicle-mediated transport

GO:0006099 tricarboxylic acid cycle

GO:0006635 fatty acid beta-oxidation

GO:0009058 biosynthetic process

GO:0006544 glycine metabolic process

GO:0006457 protein folding

GO:0007165 signal transduction

GO:0006413 translational initiation

GO:0046686 response to cadmium ion

GO:0019344 cysteine biosynthetic process

GO:0006412 translation

GO:0055114 oxidation reduction

GO:0006511 ubiquitin-dependent protein catabolic process

GO:0006633 fatty acid biosynthetic process

GO:0010388 cullin deneddylation

GO:0006563 L-serine metabolic process

GO:0006556 S-adenosylmethionine biosynthetic process

GO:0006886 intracellular protein transport

GO:0006508 proteolysis

GO:0009640 photomorphogenesis

GO:0009742 brassinosteroid mediated signaling

GO:0006995 cellular response to nitrogen starvation

GO:0006888 ER to Golgi vesicle-mediated transport

Enriched GO BP terms in the total network but not in any subnetworks:

GO:0006289 nucleotide-excision repair

GO:0009451 RNA modification

GO:0030148 sphingolipid biosynthetic process

GO:0006334 nucleosome assembly

GO:0048364 root development

GO:0006817 phosphate transport

GO:0006790 sulfur metabolic process

GO:0006396 RNA processing

GO:0006121 "mitochondrial electron transport, succinate to ubiquinone"

GO:0007169 transmembrane receptor protein tyrosine kinase signaling pathway

GO:0006874 cellular calcium ion homeostasis

GO:0006464 protein modification process

GO:0009638 phototropism

GO:0030001 metal ion transport

GO:0009736 cytokinin mediated signaling

GO:0006301 postreplication repair

GO:0008272 sulfate transport

GO:0008380 RNA splicing

GO:0009966 regulation of signal transduction

GO:0045449 regulation of transcription

GO:0009617 response to bacterium

GO:0051726 regulation of cell cycle

GO:0006891 intra-Golgi vesicle-mediated transport

GO:0006298 mismatch repair

GO:0010216 maintenance of DNA methylation

GO:0007015 actin filament organization

GO:0006828 manganese ion transport

GO:0010020 chloroplast fission

GO:0006730 one-carbon compound metabolic process

GO:0006617 "SRP-dependent cotranslational protein targeting to membrane, signal sequence recognition"

GO:0006367 transcription initiation from RNA polymerase II promoter

GO:0006510 ATP-dependent proteolysis

GO:0009616 virus induced gene silencing

GO:0006355 "regulation of transcription, DNA-dependent"

GO:0010224 response to UV-B

GO:0010617 circadian regulation of calcium ion oscillation

GO:0006875 cellular metal ion homeostasis

Root-specific enriched GO BP terms in roots network:

GO:0006519 cellular amino acid and derivative metabolic process

GO:0019509 methionine salvage

GO:0015914 phospholipid transport

GO:0015992 proton transport

GO:0009845 seed germination

Enriched GO BP terms in roots network but not total network:

GO:0009116 nucleoside metabolic process

GO:0007021 tubulin complex assembly

GO:0009156 ribonucleoside monophosphate biosynthetic process

GO:0007033 vacuole organization

GO:0008333 endosome to lysosome transport

GO:0009165 nucleotide biosynthetic process

GO:0006378 mRNA polyadenylation

GO:0045454 cell redox homeostasis

GO:0015865 purine nucleotide transport

GO:0044249 cellular biosynthetic process

GO:0050832 defense response to fungus

GO:0016036 cellular response to phosphate starvation

GO:0009845 seed germination

Leaf-specific enriched GO BP terms in leaves network:

GO:0009733 response to auxin stimulus

GO:0006529 asparagine biosynthetic process

GO:0006535 cysteine biosynthetic process from serine

GO:0019253 reductive pentose-phosphate cycle

Enriched GO BP terms in leaves network but not total network:

GO:0000103 sulfate assimilation

GO:0009744 response to sucrose stimulus

GO:0007033 vacuole organization

GO:0009733 response to auxin stimulus

GO:0000302 response to reactive oxygen species

GO:0006529 asparagine biosynthetic process

GO:0048366 leaf development

GO:0006378 mRNA polyadenylation

GO:0045454 cell redox homeostasis

GO:0009098 leucine biosynthetic process

GO:0055072 iron ion homeostasis

GO:0019253 reductive pentose-phosphate cycle

GO:0009416 response to light stimulus

GO:0015865 purine nucleotide transport

GO:0016036 cellular response to phosphate starvation

Flower-specific enriched GO BP terms in flowers network:

GO:0030048 actin filament-based movement

GO:0010267 "RNA interference, production of ta-siRNAs"

GO:0009910 negative regulation of flower development

GO:0006499 N-terminal protein myristoylation

GO:0006397 mRNA processing

GO:0006306 DNA methylation

GO:0009750 response to fructose stimulus

GO:0009850 auxin metabolic process

GO:0009738 abscisic acid mediated signaling

GO:0006221 pyrimidine nucleotide biosynthetic process

GO:0006468 protein amino acid phosphorylation

GO:0007010 cytoskeleton organization

GO:0016575 histone deacetylation

GO:0009218 pyrimidine ribonucleotide metabolic process

GO:0019761 glucosinolate biosynthetic process

GO:0006751 glutathione catabolic process

Flower-specific enriched GO BP terms not in roots/leaves network:

GO:0030048 actin filament-based movement

GO:0006268 DNA unwinding during replication

GO:0010267 "RNA interference, production of ta-siRNAs"

GO:0009910 negative regulation of flower development

GO:0044237 cellular metabolic process

GO:0045941 positive regulation of transcription

GO:0006499 N-terminal protein myristoylation

GO:0006979 response to oxidative stress

GO:0009723 response to ethylene stimulus

GO:0006397 mRNA processing

GO:0006306 DNA methylation

GO:0009750 response to fructose stimulus

GO:0009850 auxin metabolic process

GO:0042938 dipeptide transport

GO:0009738 abscisic acid mediated signaling

GO:0006013 mannose metabolic process

GO:0006221 pyrimidine nucleotide biosynthetic process

GO:0006468 protein amino acid phosphorylation

GO:0006270 DNA replication initiation

GO:0007010 cytoskeleton organization

GO:0009081 branched chain family amino acid metabolic process

GO:0000059 "protein import into nucleus, docking"

GO:0016575 histone deacetylation

GO:0009218 pyrimidine ribonucleotide metabolic process

GO:0009396 folic acid and derivative biosynthetic process

GO:0008283 cell proliferation

GO:0009809 lignin biosynthetic process

GO:0009411 response to UV

GO:0019761 glucosinolate biosynthetic process

GO:0019243 methylglyoxal catabolic process to D-lactate

GO:0006751 glutathione catabolic process

Enriched GO BP terms in flowers network but not total network:

GO:0030048 actin filament-based movement

GO:0007021 tubulin complex assembly

GO:0007033 vacuole organization

GO:0006397 mRNA processing

GO:0006306 DNA methylation

GO:0009750 response to fructose stimulus

GO:0009098 leucine biosynthetic process

GO:0016575 histone deacetylation

GO:0050832 defense response to fungus

GO:0016036 cellular response to phosphate starvation

GO:0019243 methylglyoxal catabolic process to D-lactate

Silique-specific enriched GO BP terms in siliques network:

GO:0006839 mitochondrial transport

GO:0008654 phospholipid biosynthetic process

GO:0006623 protein targeting to vacuole

GO:0006021 inositol biosynthetic process

GO:0016126 sterol biosynthetic process

GO:0031087 deadenylation-independent decapping of nuclear-transcribed mRNA

GO:0009624 response to nematode

GO:0009067 aspartate family amino acid biosynthetic process

GO:0051276 chromosome organization

Silique-specific enriched GO BP terms not in roots/leaves network:

GO:0006268 DNA unwinding during replication

GO:0006839 mitochondrial transport

GO:0008654 phospholipid biosynthetic process

GO:0006623 protein targeting to vacuole

GO:0044237 cellular metabolic process

GO:0042938 dipeptide transport

GO:0006021 inositol biosynthetic process

GO:0006013 mannose metabolic process

GO:0016126 sterol biosynthetic process

GO:0031087 deadenylation-independent decapping of nuclear-transcribed mRNA

GO:0006270 DNA replication initiation

GO:0009624 response to nematode

GO:0009081 branched chain family amino acid metabolic process

GO:0009067 aspartate family amino acid biosynthetic process

GO:0009396 folic acid and derivative biosynthetic process

GO:0008283 cell proliferation

GO:0009809 lignin biosynthetic process

GO:0009411 response to UV

GO:0019243 methylglyoxal catabolic process to D-lactate

GO:0051276 chromosome organization

Enriched GO BP terms in siliques network but not total network:

GO:0000103 sulfate assimilation

GO:0009116 nucleoside metabolic process

GO:0008654 phospholipid biosynthetic process

GO:0006623 protein targeting to vacuole

GO:0009156 ribonucleoside monophosphate biosynthetic process

GO:0009744 response to sucrose stimulus

GO:0007033 vacuole organization

GO:0008333 endosome to lysosome transport

GO:0009165 nucleotide biosynthetic process

GO:0006378 mRNA polyadenylation

GO:0006021 inositol biosynthetic process

GO:0009624 response to nematode

GO:0009067 aspartate family amino acid biosynthetic process

GO:0015865 purine nucleotide transport

GO:0044249 cellular biosynthetic process

GO:0050832 defense response to fungus

GO:0016036 cellular response to phosphate starvation

GO:0019243 methylglyoxal catabolic process to D-lactate

Seed-specific enriched GO BP terms in seeds network:

GO:0030036 actin cytoskeleton organization

GO:0006139 "nucleobase, nucleoside, nucleotide and nucleic acid metabolic process"

GO:0010188 response to microbial phytotoxin

GO:0008295 spermidine biosynthetic process

Seed-specific enriched GO BP terms not in roots/leaves network:

GO:0030036 actin cytoskeleton organization

GO:0045941 positive regulation of transcription

GO:0006979 response to oxidative stress

GO:0009723 response to ethylene stimulus

GO:0042938 dipeptide transport

GO:0006013 mannose metabolic process

GO:0006139 "nucleobase, nucleoside, nucleotide and nucleic acid metabolic process"

GO:0010188 response to microbial phytotoxin

GO:0000059 "protein import into nucleus, docking"

GO:0008295 spermidine biosynthetic process

Enriched GO BP terms in seeds network but not total network:

GO:0030036 actin cytoskeleton organization

GO:0007033 vacuole organization

GO:0000302 response to reactive oxygen species

GO:0008333 endosome to lysosome transport

GO:0048366 leaf development

GO:0055072 iron ion homeostasis

GO:0006139 "nucleobase, nucleoside, nucleotide and nucleic acid metabolic process"

GO:0009416 response to light stimulus

GO:0015865 purine nucleotide transport

GO:0016036 cellular response to phosphate starvation

GO:0008295 spermidine biosynthetic process
