# Supplementary material for: Investigating the validity of current network analysis on static conglomerate networks by protein network stratification
Source: BMC Bioinformatics. 2010 Sep 16;11:466. doi: 10.1186/1471-2105-11-466 (PMC2949894; doi:10.1186/1471-2105-11-466)
Supplement: Additional file 4 — Results of analyses using A. thaliana networks with replaced interactions. Degree distributions were drawn in figures for each of the networks with replaced interactions. Network statistics including degree, clustering coefficient, eccentricity and betweenness were calculated and compared. Network hub/bottleneck status changes were investigated. The results are generally consistent with those based on original networks. [file 1471-2105-11-466-S4.DOC]

**Additional file 4**

**Analysis Using *A. thaliana* Networks with Replaced and Additional Interactions**

***Degree distributions for unstratified and stratified networks***

| Network | Average degree | Degree exponent γ |
| --- | --- | --- |
| Total network, 10% replaced interactions | 6.842 | 2.14 |
| Stratified flowers network | 6.225 | 2.11 |
| Stratified leaves network | 4.68 | 2.18 |
| Stratified roots network | 5.937 | 2.05 |
| Stratified seeds network | 4.55 | 2.13 |
| Stratified siliques network | 5.437 | 2.13 |
|  |  |  |
| Total network, 20% replaced interactions | 6.828 | 2.22 |
| Stratified flowers network | 6.052 | 2.27 |
| Stratified leaves network | 4.393 | 2.25 |
| Stratified roots network | 5.646 | 2.2 |
| Stratified seeds network | 4.273 | 2.18 |
| Stratified siliques network | 5.224 | 2.26 |
|  |  |  |
| Total network, 30% replaced interactions | 6.798 | 2.39 |
| Stratified flowers network | 5.876 | 2.29 |
| Stratified leaves network | 4.206 | 2.35 |
| Stratified roots network | 5.413 | 2.22 |
| Stratified seeds network | 4.001 | 2.44 |
| Stratified siliques network | 4.926 | 2.39 |
|  |  |  |
| Total network, 40% replaced interactions | 6.781 | 2.45 |
| Stratified flowers network | 5.758 | 2.41 |
| Stratified leaves network | 3.94 | 2.52 |
| Stratified roots network | 5.079 | 2.42 |
| Stratified seeds network | 3.806 | 2.53 |
| Stratified siliques network | 4.736 | 2.47 |
|  |  |  |
| Total network, 10% additional interactions | 7.382 | 2.08 |
| Stratified flowers network | 6.718 | 2.04 |
| Stratified leaves network | 5.026 | 2.02 |
| Stratified roots network | 6.471 | 1.96 |
| Stratified seeds network | 4.892 | 2.09 |
| Stratified siliques network | 5.907 | 2.13 |
|  |  |  |
| Total network, 20% additional interactions | 8.053 | 2.12 |
| Stratified flowers network | 7.135 | 2.08 |
| Stratified leaves network | 5.134 | 2.2 |
| Stratified roots network | 6.644 | 2.02 |
| Stratified seeds network | 4.972 | 2.17 |
| Stratified siliques network | 6.109 | 2.12 |

***Network Statistics are Different between the Total Network and Stratified Subnetworks***

For networks with 10% replaced interactions:

| Degree | cell_culture_10pfnfp_stat.txt | flowers_10pfnfn_stat.txt | leaves_10pfnfp_stat.txt | roots_10pfnfp_stat.txt | seeds_10pfnfp_stat.txt | siliques_10pfnfp_stat.txt | total_10pfnfp_stat.txt |
| --- | --- | --- | --- | --- | --- | --- | --- |
| cell_culture_10pfnfp_stat.txt | 1 | 0.000711 | 1.94E-30 | 3.19E-06 | 5.55E-33 | 1.17E-13 | 0.893596 |
| flowers_10pfnfn_stat.txt | 0.000711 | 1 | 7.90E-20 | 0.108954 | 3.18E-22 | 4.59E-06 | 0.000211 |
| leaves_10pfnfp_stat.txt | 1.94E-30 | 7.90E-20 | 1 | 3.33E-12 | 0.448614 | 1.10E-05 | 5.15E-38 |
| roots_10pfnfp_stat.txt | 3.19E-06 | 0.108954 | 3.33E-12 | 1 | 4.08E-14 | 0.006287 | 3.61E-07 |
| seeds_10pfnfp_stat.txt | 5.55E-33 | 3.18E-22 | 0.448614 | 4.08E-14 | 1 | 4.15E-07 | 7.18E-41 |
| siliques_10pfnfp_stat.txt | 1.17E-13 | 4.59E-06 | 1.10E-05 | 0.006287 | 4.15E-07 | 1 | 1.39E-16 |
| total_10pfnfp_stat.txt | 0.893596 | 0.000211 | 5.15E-38 | 3.61E-07 | 7.18E-41 | 1.39E-16 | 1 |
|  |  |  |  |  |  |  |  |
|  |  |  |  |  |  |  |  |
| Clu. Coeffy | cell_culture_10pfnfp_stat.txt | flowers_10pfnfn_stat.txt | leaves_10pfnfp_stat.txt | roots_10pfnfp_stat.txt | seeds_10pfnfp_stat.txt | siliques_10pfnfp_stat.txt | total_10pfnfp_stat.txt |
| cell_culture_10pfnfp_stat.txt | 1 | 3.60E-06 | 0.001984 | 0.000141 | 0.000373 | 1.52E-06 | 0.000246 |
| flowers_10pfnfn_stat.txt | 3.60E-06 | 1 | 0.62365 | 0.713618 | 0.814787 | 0.428848 | 0.155955 |
| leaves_10pfnfp_stat.txt | 0.001984 | 0.62365 | 1 | 0.870331 | 0.550469 | 0.271697 | 0.583362 |
| roots_10pfnfp_stat.txt | 0.000141 | 0.713618 | 0.870331 | 1 | 0.616824 | 0.28933 | 0.386537 |
| seeds_10pfnfp_stat.txt | 0.000373 | 0.814787 | 0.550469 | 0.616824 | 1 | 0.693543 | 0.219506 |
| siliques_10pfnfp_stat.txt | 1.52E-06 | 0.428848 | 0.271697 | 0.28933 | 0.693543 | 1 | 0.04013 |
| total_10pfnfp_stat.txt | 0.000246 | 0.155955 | 0.583362 | 0.386537 | 0.219506 | 0.04013 | 1 |
|  |  |  |  |  |  |  |  |
|  |  |  |  |  |  |  |  |
| Betweenness | cell_culture_10pfnfp_stat.txt | flowers_10pfnfn_stat.txt | leaves_10pfnfp_stat.txt | roots_10pfnfp_stat.txt | seeds_10pfnfp_stat.txt | siliques_10pfnfp_stat.txt | total_10pfnfp_stat.txt |
| cell_culture_10pfnfp_stat.txt | 1 | 0.000117 | 1.21E-35 | 2.06E-08 | 1.46E-60 | 1.51E-10 | 2.43E-36 |
| flowers_10pfnfn_stat.txt | 0.000117 | 1 | 1.23E-52 | 1.04E-19 | 3.38E-78 | 4.98E-23 | 4.30E-18 |
| leaves_10pfnfp_stat.txt | 1.21E-35 | 1.23E-52 | 1 | 1.43E-12 | 0.000246 | 9.83E-12 | 8.30E-116 |
| roots_10pfnfp_stat.txt | 2.06E-08 | 1.04E-19 | 1.43E-12 | 1 | 1.76E-28 | 0.533439 | 7.96E-68 |
| seeds_10pfnfp_stat.txt | 1.46E-60 | 3.38E-78 | 0.000246 | 1.76E-28 | 1 | 9.82E-29 | 3.62E-148 |
| siliques_10pfnfp_stat.txt | 1.51E-10 | 4.98E-23 | 9.83E-12 | 0.533439 | 9.82E-29 | 1 | 2.68E-74 |
| total_10pfnfp_stat.txt | 2.43E-36 | 4.30E-18 | 8.30E-116 | 7.96E-68 | 3.62E-148 | 2.68E-74 | 1 |
|  |  |  |  |  |  |  |  |
|  |  |  |  |  |  |  |  |
| Eccentricity | cell_culture_10pfnfp_stat.txt | flowers_10pfnfn_stat.txt | leaves_10pfnfp_stat.txt | roots_10pfnfp_stat.txt | seeds_10pfnfp_stat.txt | siliques_10pfnfp_stat.txt | total_10pfnfp_stat.txt |
| cell_culture_10pfnfp_stat.txt | 1 | 2.55E-155 | 1.74E-113 | 1.09E-10 | 4.75E-109 | 4.76E-20 | 0 |
| flowers_10pfnfn_stat.txt | 2.55E-155 | 1 | 0 | 4.13E-185 | 1.20E-303 | 2.45E-34 | 6.10E-280 |
| leaves_10pfnfp_stat.txt | 1.74E-113 | 0 | 1 | 3.96E-57 | 0.059984 | 2.63E-154 | 0 |
| roots_10pfnfp_stat.txt | 1.09E-10 | 4.13E-185 | 3.96E-57 | 1 | 1.23E-60 | 1.12E-41 | 0 |
| seeds_10pfnfp_stat.txt | 4.75E-109 | 1.20E-303 | 0.059984 | 1.23E-60 | 1 | 7.76E-150 | 0 |
| siliques_10pfnfp_stat.txt | 4.76E-20 | 2.45E-34 | 2.63E-154 | 1.12E-41 | 7.76E-150 | 1 | 0 |
| total_10pfnfp_stat.txt | 0 | 6.10E-280 | 0 | 0 | 0 | 0 | 1 |

For networks with 20% replaced interactions:

| Degree | cell_culture_20pfnfp_stat.txt | flowers_20pfnfn_stat.txt | leaves_20pfnfp_stat.txt | roots_20pfnfp_stat.txt | seeds_20pfnfp_stat.txt | siliques_20pfnfp_stat.txt | total_20pfnfp_stat.txt |
| --- | --- | --- | --- | --- | --- | --- | --- |
| cell_culture_20pfnfp_stat.txt | 1 | 0.003769 | 1.02E-37 | 3.86E-07 | 2.71E-40 | 8.12E-15 | 0.079449 |
| flowers_20pfnfn_stat.txt | 0.003769 | 1 | 1.25E-28 | 0.010809 | 3.52E-31 | 5.07E-08 | 1.76E-07 |
| leaves_20pfnfp_stat.txt | 1.02E-37 | 1.25E-28 | 1 | 2.17E-15 | 0.427263 | 3.35E-08 | 1.31E-60 |
| roots_20pfnfp_stat.txt | 3.86E-07 | 0.010809 | 2.17E-15 | 1 | 1.81E-17 | 0.008502 | 6.51E-14 |
| seeds_20pfnfp_stat.txt | 2.71E-40 | 3.52E-31 | 0.427263 | 1.81E-17 | 1 | 6.70E-10 | 3.31E-63 |
| siliques_20pfnfp_stat.txt | 8.12E-15 | 5.07E-08 | 3.35E-08 | 0.008502 | 6.70E-10 | 1 | 1.57E-26 |
| total_20pfnfp_stat.txt | 0.079449 | 1.76E-07 | 1.31E-60 | 6.51E-14 | 3.31E-63 | 1.57E-26 | 1 |
|  |  |  |  |  |  |  |  |
|  |  |  |  |  |  |  |  |
| Clus. Coeffy | cell_culture_20pfnfp_stat.txt | flowers_20pfnfn_stat.txt | leaves_20pfnfp_stat.txt | roots_20pfnfp_stat.txt | seeds_20pfnfp_stat.txt | siliques_20pfnfp_stat.txt | total_20pfnfp_stat.txt |
| cell_culture_20pfnfp_stat.txt | 1 | 5.16E-05 | 0.002274 | 0.00012 | 0.00188 | 8.02E-06 | 0.005332 |
| flowers_20pfnfn_stat.txt | 5.16E-05 | 1 | 0.973717 | 0.808349 | 0.808723 | 0.317971 | 0.092119 |
| leaves_20pfnfp_stat.txt | 0.002274 | 0.973717 | 1 | 0.821415 | 0.814544 | 0.398586 | 0.228202 |
| roots_20pfnfp_stat.txt | 0.00012 | 0.808349 | 0.821415 | 1 | 0.964264 | 0.481779 | 0.082833 |
| seeds_20pfnfp_stat.txt | 0.00188 | 0.808723 | 0.814544 | 0.964264 | 1 | 0.586377 | 0.162 |
| siliques_20pfnfp_stat.txt | 8.02E-06 | 0.317971 | 0.398586 | 0.481779 | 0.586377 | 1 | 0.013202 |
| total_20pfnfp_stat.txt | 0.005332 | 0.092119 | 0.228202 | 0.082833 | 0.162 | 0.013202 | 1 |
|  |  |  |  |  |  |  |  |
|  |  |  |  |  |  |  |  |
| Betweenness | cell_culture_20pfnfp_stat.txt | flowers_20pfnfn_stat.txt | leaves_20pfnfp_stat.txt | roots_20pfnfp_stat.txt | seeds_20pfnfp_stat.txt | siliques_20pfnfp_stat.txt | total_20pfnfp_stat.txt |
| cell_culture_20pfnfp_stat.txt | 1 | 0.001284 | 1.16E-21 | 1.44E-05 | 2.03E-44 | 1.28E-06 | 2.84E-25 |
| flowers_20pfnfn_stat.txt | 0.001284 | 1 | 2.79E-34 | 2.58E-13 | 9.30E-60 | 2.83E-15 | 2.11E-12 |
| leaves_20pfnfp_stat.txt | 1.16E-21 | 2.79E-34 | 1 | 2.08E-07 | 9.46E-05 | 1.45E-07 | 3.55E-80 |
| roots_20pfnfp_stat.txt | 1.44E-05 | 2.58E-13 | 2.08E-07 | 1 | 6.50E-21 | 0.783687 | 2.48E-46 |
| seeds_20pfnfp_stat.txt | 2.03E-44 | 9.30E-60 | 9.46E-05 | 6.50E-21 | 1 | 4.24E-23 | 6.05E-117 |
| siliques_20pfnfp_stat.txt | 1.28E-06 | 2.83E-15 | 1.45E-07 | 0.783687 | 4.24E-23 | 1 | 2.80E-51 |
| total_20pfnfp_stat.txt | 2.84E-25 | 2.11E-12 | 3.55E-80 | 2.48E-46 | 6.05E-117 | 2.80E-51 | 1 |
|  |  |  |  |  |  |  |  |
|  |  |  |  |  |  |  |  |
| Eccentricity | cell_culture_20pfnfp_stat.txt | flowers_20pfnfn_stat.txt | leaves_20pfnfp_stat.txt | roots_20pfnfp_stat.txt | seeds_20pfnfp_stat.txt | siliques_20pfnfp_stat.txt | total_20pfnfp_stat.txt |
| cell_culture_20pfnfp_stat.txt | 1 | 0.001339 | 4.80E-22 | 3.82E-133 | 8.87E-16 | 4.66E-115 | 1.07E-157 |
| flowers_20pfnfn_stat.txt | 0.001339 | 1 | 1.03E-15 | 1.43E-170 | 2.30E-11 | 1.21E-148 | 8.96E-224 |
| leaves_20pfnfp_stat.txt | 4.80E-22 | 1.03E-15 | 1 | 9.98E-124 | 0.904965 | 7.89E-115 | 2.73E-112 |
| roots_20pfnfp_stat.txt | 3.82E-133 | 1.43E-170 | 9.98E-124 | 1 | 1.03E-86 | 0.228787 | 3.39E-09 |
| seeds_20pfnfp_stat.txt | 8.87E-16 | 2.30E-11 | 0.904965 | 1.03E-86 | 1 | 6.78E-81 | 4.15E-75 |
| siliques_20pfnfp_stat.txt | 4.66E-115 | 1.21E-148 | 7.89E-115 | 0.228787 | 6.78E-81 | 1 | 2.33E-05 |
| total_20pfnfp_stat.txt | 1.07E-157 | 8.96E-224 | 2.73E-112 | 3.39E-09 | 4.15E-75 | 2.33E-05 | 1 |

For networks with 30% replaced interactions:

| Degree | cell_culture_30pfnfp_stat.txt | flowers_30pfnfn_stat.txt | leaves_30pfnfp_stat.txt | roots_30pfnfp_stat.txt | seeds_30pfnfp_stat.txt | siliques_30pfnfp_stat.txt | total_30pfnfp_stat.txt |
| --- | --- | --- | --- | --- | --- | --- | --- |
| cell_culture_30pfnfp_stat.txt | 1 | 0.005154 | 7.80E-45 | 1.70E-08 | 2.93E-52 | 6.64E-20 | 0.000513 |
| flowers_30pfnfn_stat.txt | 0.005154 | 1 | 2.10E-36 | 0.00099 | 6.47E-44 | 1.22E-12 | 2.25E-12 |
| leaves_30pfnfp_stat.txt | 7.80E-45 | 2.10E-36 | 1 | 9.09E-18 | 0.125244 | 6.54E-08 | 7.17E-85 |
| roots_30pfnfp_stat.txt | 1.70E-08 | 0.00099 | 9.09E-18 | 1 | 4.41E-23 | 0.000602 | 5.07E-23 |
| seeds_30pfnfp_stat.txt | 2.93E-52 | 6.47E-44 | 0.125244 | 4.41E-23 | 1 | 8.45E-12 | 1.05E-94 |
| siliques_30pfnfp_stat.txt | 6.64E-20 | 1.22E-12 | 6.54E-08 | 0.000602 | 8.45E-12 | 1 | 1.18E-44 |
| total_30pfnfp_stat.txt | 0.000513 | 2.25E-12 | 7.17E-85 | 5.07E-23 | 1.05E-94 | 1.18E-44 | 1 |
|  |  |  |  |  |  |  |  |
|  |  |  |  |  |  |  |  |
| Clus. Coeffy | cell_culture_30pfnfp_stat.txt | flowers_30pfnfn_stat.txt | leaves_30pfnfp_stat.txt | roots_30pfnfp_stat.txt | seeds_30pfnfp_stat.txt | siliques_30pfnfp_stat.txt | total_30pfnfp_stat.txt |
| cell_culture_30pfnfp_stat.txt | 1 | 0.001098 | 5.37E-05 | 4.90E-07 | 6.68E-10 | 4.95E-06 | 0.080714 |
| flowers_30pfnfn_stat.txt | 0.001098 | 1 | 0.1066 | 0.02715 | 3.15E-05 | 0.071463 | 0.066689 |
| leaves_30pfnfp_stat.txt | 5.37E-05 | 0.1066 | 1 | 0.779983 | 0.013254 | 0.984824 | 0.002653 |
| roots_30pfnfp_stat.txt | 4.90E-07 | 0.02715 | 0.779983 | 1 | 0.015678 | 0.741174 | 9.44E-05 |
| seeds_30pfnfp_stat.txt | 6.68E-10 | 3.15E-05 | 0.013254 | 0.015678 | 1 | 0.008061 | 7.86E-08 |
| siliques_30pfnfp_stat.txt | 4.95E-06 | 0.071463 | 0.984824 | 0.741174 | 0.008061 | 1 | 0.000598 |
| total_30pfnfp_stat.txt | 0.080714 | 0.066689 | 0.002653 | 9.44E-05 | 7.86E-08 | 0.000598 | 1 |
|  |  |  |  |  |  |  |  |
|  |  |  |  |  |  |  |  |
| Betweenness | cell_culture_30pfnfp_stat.txt | flowers_30pfnfn_stat.txt | leaves_30pfnfp_stat.txt | roots_30pfnfp_stat.txt | seeds_30pfnfp_stat.txt | siliques_30pfnfp_stat.txt | total_30pfnfp_stat.txt |
| cell_culture_30pfnfp_stat.txt | 1 | 0.001264 | 4.19E-23 | 5.62E-05 | 2.10E-40 | 2.66E-07 | 7.00E-22 |
| flowers_30pfnfn_stat.txt | 0.001264 | 1 | 5.91E-37 | 1.21E-12 | 2.15E-57 | 1.52E-16 | 3.24E-10 |
| leaves_30pfnfp_stat.txt | 4.19E-23 | 5.91E-37 | 1 | 3.54E-09 | 0.003048 | 2.13E-07 | 4.25E-79 |
| roots_30pfnfp_stat.txt | 5.62E-05 | 1.21E-12 | 3.54E-09 | 1 | 4.53E-20 | 0.327289 | 5.55E-41 |
| seeds_30pfnfp_stat.txt | 2.10E-40 | 2.15E-57 | 0.003048 | 4.53E-20 | 1 | 5.31E-18 | 1.39E-108 |
| siliques_30pfnfp_stat.txt | 2.66E-07 | 1.52E-16 | 2.13E-07 | 0.327289 | 5.31E-18 | 1 | 1.00E-48 |
| total_30pfnfp_stat.txt | 7.00E-22 | 3.24E-10 | 4.25E-79 | 5.55E-41 | 1.39E-108 | 1.00E-48 | 1 |
|  |  |  |  |  |  |  |  |
|  |  |  |  |  |  |  |  |
| Eccentricity | cell_culture_30pfnfp_stat.txt | flowers_30pfnfn_stat.txt | leaves_30pfnfp_stat.txt | roots_30pfnfp_stat.txt | seeds_30pfnfp_stat.txt | siliques_30pfnfp_stat.txt | total_30pfnfp_stat.txt |
| cell_culture_30pfnfp_stat.txt | 1 | 0 | 4.55E-09 | 1.78E-25 | 0 | 4.57E-18 | 4.51E-45 |
| flowers_30pfnfn_stat.txt | 0 | 1 | 2.12E-203 | 0 | 9.43E-233 | 0 | 0 |
| leaves_30pfnfp_stat.txt | 4.55E-09 | 2.12E-203 | 1 | 0.536737 | 0 | 0.6292 | 6.62E-35 |
| roots_30pfnfp_stat.txt | 1.78E-25 | 0 | 0.536737 | 1 | 0 | 0.894604 | 4.43E-110 |
| seeds_30pfnfp_stat.txt | 0 | 9.43E-233 | 0 | 0 | 1 | 0 | 0 |
| siliques_30pfnfp_stat.txt | 4.57E-18 | 0 | 0.6292 | 0.894604 | 0 | 1 | 8.32E-73 |
| total_30pfnfp_stat.txt | 4.51E-45 | 0 | 6.62E-35 | 4.43E-110 | 0 | 8.32E-73 | 1 |

For networks with 40% replaced interactions:

| Degree | flowers_40pfnfp_stat.txt | leaves_40pfnfp_stat.txt | roots_40pfnfp_stat.txt | seeds_40pfnfp_stat.txt | siliques_40pfnfp_stat.txt | total_40pfnfp_stat.txt |
| --- | --- | --- | --- | --- | --- | --- |
| flowers_40pfnfp_stat.txt | 1 | 1.06E-56 | 1.57E-08 | 1.87E-60 | 7.85E-19 | 2.77E-19 |
| leaves_40pfnfp_stat.txt | 1.06E-56 | 1 | 1.90E-21 | 0.252397 | 3.81E-12 | 1.38E-133 |
| roots_40pfnfp_stat.txt | 1.57E-08 | 1.90E-21 | 1 | 8.47E-25 | 0.004516 | 1.87E-45 |
| seeds_40pfnfp_stat.txt | 1.87E-60 | 0.252397 | 8.47E-25 | 1 | 4.92E-15 | 7.07E-135 |
| siliques_40pfnfp_stat.txt | 7.85E-19 | 3.81E-12 | 0.004516 | 4.92E-15 | 1 | 6.28E-70 |
| total_40pfnfp_stat.txt | 2.77E-19 | 1.38E-133 | 1.87E-45 | 7.07E-135 | 6.28E-70 | 1 |
|  |  |  |  |  |  |  |
|  |  |  |  |  |  |  |
| Clustering coefficient | flowers_40pfnfp_stat.txt | leaves_40pfnfp_stat.txt | roots_40pfnfp_stat.txt | seeds_40pfnfp_stat.txt | siliques_40pfnfp_stat.txt | total_40pfnfp_stat.txt |
| flowers_40pfnfp_stat.txt | 1 | 0.088158 | 0.550151 | 7.84E-06 | 0.217136 | 0.011039 |
| leaves_40pfnfp_stat.txt | 0.088158 | 1 | 0.260506 | 0.007267 | 0.553595 | 0.000536 |
| roots_40pfnfp_stat.txt | 0.550151 | 0.260506 | 1 | 0.00011 | 0.552724 | 0.005824 |
| seeds_40pfnfp_stat.txt | 7.84E-06 | 0.007267 | 0.00011 | 1 | 0.00072 | 2.95E-09 |
| siliques_40pfnfp_stat.txt | 0.217136 | 0.553595 | 0.552724 | 0.00072 | 1 | 0.00084 |
| total_40pfnfp_stat.txt | 0.011039 | 0.000536 | 0.005824 | 2.95E-09 | 0.00084 | 1 |
|  |  |  |  |  |  |  |
|  |  |  |  |  |  |  |
| Betweenness | flowers_40pfnfp_stat.txt | leaves_40pfnfp_stat.txt | roots_40pfnfp_stat.txt | seeds_40pfnfp_stat.txt | siliques_40pfnfp_stat.txt | total_40pfnfp_stat.txt |
| flowers_40pfnfp_stat.txt | 1 | 7.66E-33 | 1.07E-10 | 1.11E-60 | 3.72E-13 | 6.49E-10 |
| leaves_40pfnfp_stat.txt | 7.66E-33 | 1 | 1.93E-08 | 3.82E-05 | 1.11E-07 | 5.92E-74 |
| roots_40pfnfp_stat.txt | 1.07E-10 | 1.93E-08 | 1 | 1.23E-23 | 0.560133 | 4.33E-37 |
| seeds_40pfnfp_stat.txt | 1.11E-60 | 3.82E-05 | 1.23E-23 | 1 | 1.32E-23 | 4.53E-116 |
| siliques_40pfnfp_stat.txt | 3.72E-13 | 1.11E-07 | 0.560133 | 1.32E-23 | 1 | 6.83E-43 |
| total_40pfnfp_stat.txt | 6.49E-10 | 5.92E-74 | 4.33E-37 | 4.53E-116 | 6.83E-43 | 1 |
|  |  |  |  |  |  |  |
|  |  |  |  |  |  |  |
| Eccentricity | flowers_40pfnfp_stat.txt | leaves_40pfnfp_stat.txt | roots_40pfnfp_stat.txt | seeds_40pfnfp_stat.txt | siliques_40pfnfp_stat.txt | total_40pfnfp_stat.txt |
| flowers_40pfnfp_stat.txt | 1 | 1.79E-225 | 3.03E-69 | 5.17E-184 | 0 | 0 |
| leaves_40pfnfp_stat.txt | 1.79E-225 | 1 | 2.84E-127 | 0.00029 | 2.75E-20 | 0 |
| roots_40pfnfp_stat.txt | 3.03E-69 | 2.84E-127 | 1 | 4.99E-117 | 3.91E-122 | 0 |
| seeds_40pfnfp_stat.txt | 5.17E-184 | 0.00029 | 4.99E-117 | 1 | 4.93E-31 | 6.8576311642765e-321 |
| siliques_40pfnfp_stat.txt | 0 | 2.75E-20 | 3.91E-122 | 4.93E-31 | 1 | 0 |
| total_40pfnfp_stat.txt | 0 | 0 | 0 | 6.8576311642765e-321 | 0 | 1 |

For networks with 10% additional interactions:

| Degree | flowers_20pfn_stat.txt | leaves_20pfn_stat.txt | roots_20pfn_stat.txt | seeds_20pfn_stat.txt | siliques_20pfn_stat.txt | total_20pfn_stat.txt |
| --- | --- | --- | --- | --- | --- | --- |
| flowers_20pfn_stat.txt | 1 | 1.59E-28 | 0.011039 | 9.17E-32 | 2.87E-08 | 4.01E-07 |
| leaves_20pfn_stat.txt | 1.59E-28 | 1 | 2.74E-15 | 0.370153 | 8.74E-08 | 3.74E-59 |
| roots_20pfn_stat.txt | 0.011039 | 2.74E-15 | 1 | 8.51E-18 | 0.006165 | 2.22E-13 |
| seeds_20pfn_stat.txt | 9.17E-32 | 0.370153 | 8.51E-18 | 1 | 9.42E-10 | 6.84E-63 |
| siliques_20pfn_stat.txt | 2.87E-08 | 8.74E-08 | 0.006165 | 9.42E-10 | 1 | 4.05E-26 |
| total_20pfn_stat.txt | 4.01E-07 | 3.74E-59 | 2.22E-13 | 6.84E-63 | 4.05E-26 | 1 |
|  |  |  |  |  |  |  |
|  |  |  |  |  |  |  |
| Clustering Coefficient | flowers_20pfn_stat.txt | leaves_20pfn_stat.txt | roots_20pfn_stat.txt | seeds_20pfn_stat.txt | siliques_20pfn_stat.txt | total_20pfn_stat.txt |
| flowers_20pfn_stat.txt | 1 | 0.695437 | 0.470452 | 0.031286 | 0.076548 | 0.102354 |
| leaves_20pfn_stat.txt | 0.695437 | 1 | 0.830027 | 0.118719 | 0.275149 | 0.103896 |
| roots_20pfn_stat.txt | 0.470452 | 0.830027 | 1 | 0.132021 | 0.319074 | 0.026487 |
| seeds_20pfn_stat.txt | 0.031286 | 0.118719 | 0.132021 | 1 | 0.510623 | 0.000843 |
| siliques_20pfn_stat.txt | 0.076548 | 0.275149 | 0.319074 | 0.510623 | 1 | 0.001085 |
| total_20pfn_stat.txt | 0.102354 | 0.103896 | 0.026487 | 0.000843 | 0.001085 | 1 |
|  |  |  |  |  |  |  |
|  |  |  |  |  |  |  |
| Betweenness | flowers_20pfn_stat.txt | leaves_20pfn_stat.txt | roots_20pfn_stat.txt | seeds_20pfn_stat.txt | siliques_20pfn_stat.txt | total_20pfn_stat.txt |
| flowers_20pfn_stat.txt | 1 | 4.53E-36 | 2.42E-12 | 6.13E-65 | 3.30E-16 | 8.26E-12 |
| leaves_20pfn_stat.txt | 4.53E-36 | 1 | 4.92E-09 | 4.89E-05 | 3.15E-08 | 6.36E-81 |
| roots_20pfn_stat.txt | 2.42E-12 | 4.92E-09 | 1 | 3.97E-25 | 0.424528 | 3.28E-43 |
| seeds_20pfn_stat.txt | 6.13E-65 | 4.89E-05 | 3.97E-25 | 1 | 1.67E-26 | 5.77E-122 |
| siliques_20pfn_stat.txt | 3.30E-16 | 3.15E-08 | 0.424528 | 1.67E-26 | 1 | 6.86E-52 |
| total_20pfn_stat.txt | 8.26E-12 | 6.36E-81 | 3.28E-43 | 5.77E-122 | 6.86E-52 | 1 |
|  |  |  |  |  |  |  |
|  |  |  |  |  |  |  |
| Eccentricity | flowers_20pfn_stat.txt | leaves_20pfn_stat.txt | roots_20pfn_stat.txt | seeds_20pfn_stat.txt | siliques_20pfn_stat.txt | total_20pfn_stat.txt |
| flowers_20pfn_stat.txt | 1 | 1.63E-19 | 2.85E-55 | 9.79E-12 | 0.038769 | 0 |
| leaves_20pfn_stat.txt | 1.63E-19 | 1 | 0.205843 | 5.64E-32 | 3.24E-21 | 8.09E-179 |
| roots_20pfn_stat.txt | 2.85E-55 | 0.205843 | 1 | 4.22E-50 | 2.10E-47 | 0 |
| seeds_20pfn_stat.txt | 9.79E-12 | 5.64E-32 | 4.22E-50 | 1 | 3.57E-07 | 3.18E-31 |
| siliques_20pfn_stat.txt | 0.038769 | 3.24E-21 | 2.10E-47 | 3.57E-07 | 1 | 3.01E-175 |
| total_20pfn_stat.txt | 0 | 8.09E-179 | 0 | 3.18E-31 | 3.01E-175 | 1 |

For networks with 20% additional interactions:

| Degree | flowers_10pfn_stat.txt | leaves_10pfn_stat.txt | roots_10pfn_stat.txt | seeds_10pfn_stat.txt | siliques_10pfn_stat.txt | total_10pfn_stat.txt |
| --- | --- | --- | --- | --- | --- | --- |
| flowers_10pfn_stat.txt | 1 | 4.75E-20 | 0.211215 | 2.38E-22 | 1.62E-05 | 0.000266 |
| leaves_10pfn_stat.txt | 4.75E-20 | 1 | 2.33E-13 | 0.472158 | 2.64E-06 | 4.10E-38 |
| roots_10pfn_stat.txt | 0.211215 | 2.33E-13 | 1 | 3.06E-15 | 0.004919 | 3.06E-06 |
| seeds_10pfn_stat.txt | 2.38E-22 | 0.472158 | 3.06E-15 | 1 | 1.02E-07 | 7.09E-41 |
| siliques_10pfn_stat.txt | 1.62E-05 | 2.64E-06 | 0.004919 | 1.02E-07 | 1 | 2.13E-15 |
| total_10pfn_stat.txt | 0.000266 | 4.10E-38 | 3.06E-06 | 7.09E-41 | 2.13E-15 | 1 |
|  |  |  |  |  |  |  |
|  |  |  |  |  |  |  |
| Clustering Coefficient | flowers_10pfn_stat.txt | leaves_10pfn_stat.txt | roots_10pfn_stat.txt | seeds_10pfn_stat.txt | siliques_10pfn_stat.txt | total_10pfn_stat.txt |
| flowers_10pfn_stat.txt | 1 | 0.635219 | 0.935087 | 0.684402 | 0.257543 | 0.398632 |
| leaves_10pfn_stat.txt | 0.635219 | 1 | 0.608597 | 0.461175 | 0.16995 | 0.88671 |
| roots_10pfn_stat.txt | 0.935087 | 0.608597 | 1 | 0.747341 | 0.328977 | 0.40078 |
| seeds_10pfn_stat.txt | 0.684402 | 0.461175 | 0.747341 | 1 | 0.613512 | 0.307369 |
| siliques_10pfn_stat.txt | 0.257543 | 0.16995 | 0.328977 | 0.613512 | 1 | 0.052913 |
| total_10pfn_stat.txt | 0.398632 | 0.88671 | 0.40078 | 0.307369 | 0.052913 | 1 |
|  |  |  |  |  |  |  |
|  |  |  |  |  |  |  |
| Betweenness | flowers_10pfn_stat.txt | leaves_10pfn_stat.txt | roots_10pfn_stat.txt | seeds_10pfn_stat.txt | siliques_10pfn_stat.txt | total_10pfn_stat.txt |
| flowers_10pfn_stat.txt | 1 | 6.86E-42 | 1.56E-15 | 1.91E-67 | 3.33E-22 | 1.25E-18 |
| leaves_10pfn_stat.txt | 6.86E-42 | 1 | 4.46E-10 | 0.000162 | 5.15E-07 | 1.73E-100 |
| roots_10pfn_stat.txt | 1.56E-15 | 4.46E-10 | 1 | 2.35E-25 | 0.110805 | 2.38E-60 |
| seeds_10pfn_stat.txt | 1.91E-67 | 0.000162 | 2.35E-25 | 1 | 1.22E-21 | 9.82E-134 |
| siliques_10pfn_stat.txt | 3.33E-22 | 5.15E-07 | 0.110805 | 1.22E-21 | 1 | 7.01E-73 |
| total_10pfn_stat.txt | 1.25E-18 | 1.73E-100 | 2.38E-60 | 9.82E-134 | 7.01E-73 | 1 |
|  |  |  |  |  |  |  |
|  |  |  |  |  |  |  |
| Eccentricity | flowers_10pfn_stat.txt | leaves_10pfn_stat.txt | roots_10pfn_stat.txt | seeds_10pfn_stat.txt | siliques_10pfn_stat.txt | total_10pfn_stat.txt |
| flowers_10pfn_stat.txt | 1 | 1.46E-09 | 0.000444 | 1.34E-85 | 9.12E-89 | 5.65E-27 |
| leaves_10pfn_stat.txt | 1.46E-09 | 1 | 0.00209 | 2.92E-30 | 1.40E-19 | 0.907131 |
| roots_10pfn_stat.txt | 0.000444 | 0.00209 | 1 | 6.85E-58 | 1.05E-49 | 8.84E-07 |
| seeds_10pfn_stat.txt | 1.34E-85 | 2.92E-30 | 6.85E-58 | 1 | 1.68E-05 | 1.35E-49 |
| siliques_10pfn_stat.txt | 9.12E-89 | 1.40E-19 | 1.05E-49 | 1.68E-05 | 1 | 1.34E-43 |
| total_10pfn_stat.txt | 5.65E-27 | 0.907131 | 8.84E-07 | 1.35E-49 | 1.34E-43 | 1 |

***Summary of Hubs Status Change between the Total Network and Stratified Subnetworks***

For networks with 10% replaced interactions:

5%: nonhub to hub in roots: 1, # of hubs in roots: 165 0.6%

5%: hub to nonhub in roots: 92, # of hubs in total: 340 27.1%

5%: nonhub to hub in leaves: 7, # of hubs in leaves: 119 5.9%

5%: hub to nonhub in leaves: 63, # of hubs in total: 340 18.5%

5%: nonhub to hub in flowers: 7, # of hubs in flowers: 247 2.8%

5%: hub to nonhub in flowers: 46, # of hubs in total: 340 13.5%

5%: nonhub to hub in siliques: 3, # of hubs in siliques: 157 1.9%

5%: hub to nonhub in siliques: 76, # of hubs in total: 340 22.4%

5%: nonhub to hub in seeds: 4, # of hubs in seeds: 87 4.6%

5%: hub to nonhub in seeds: 78, # of hubs in total: 340 22.9%

10%: nonhub to hub in roots: 1, # of hubs in roots: 323 0.3%

10%: hub to nonhub in roots: 139, # of hubs in total: 647 21.5%

10%: nonhub to hub in leaves: 9, # of hubs in leaves: 222 4.1%

10%: hub to nonhub in leaves: 120, # of hubs in total: 647 18.5%

10%: nonhub to hub in flowers: 14, # of hubs in flowers: 479 2.9%

10%: hub to nonhub in flowers: 65, # of hubs in total: 647 10.0%

10%: nonhub to hub in siliques: 16, # of hubs in siliques: 328 4.9%

10%: hub to nonhub in siliques: 98, # of hubs in total: 647 15.1%

10%: nonhub to hub in seeds: 9, # of hubs in seeds: 200 4.5%

10%: hub to nonhub in seeds: 92, # of hubs in total: 647 14.2%

20%: nonhub to hub in roots: 6, # of hubs in roots: 648 0.9%

20%: hub to nonhub in roots: 216, # of hubs in total: 1268 17.0%

20%: nonhub to hub in leaves: 9, # of hubs in leaves: 421 2.1%

20%: hub to nonhub in leaves: 208, # of hubs in total: 1268 16.4%

20%: nonhub to hub in flowers: 0, # of hubs in flowers: 882 0%

20%: hub to nonhub in flowers: 174, # of hubs in total: 1268 13.7%

20%: nonhub to hub in siliques: 19, # of hubs in siliques: 635 3.0%

20%: hub to nonhub in siliques: 171, # of hubs in total: 1268 13.5%

20%: nonhub to hub in seeds: 13, # of hubs in seeds: 366 3.6%

20%: hub to nonhub in seeds: 187, # of hubs in total: 1268 14.7%

For networks with 20% replaced interactions:

5%: nonhub to hub in roots: 0, # of hubs in roots: 170 0%

5%: hub to nonhub in roots: 78, # of hubs in total: 336 23.2%

5%: nonhub to hub in leaves: 13, # of hubs in leaves: 122 10.7%

5%: hub to nonhub in leaves: 66, # of hubs in total: 336 19.6%

5%: nonhub to hub in flowers: 2, # of hubs in flowers: 229 0.9%

5%: hub to nonhub in flowers: 56, # of hubs in total: 336 16.7%

5%: nonhub to hub in siliques: 7, # of hubs in siliques: 163 4.3%

5%: hub to nonhub in siliques: 72, # of hubs in total: 336 21.4%

5%: nonhub to hub in seeds: 2, # of hubs in seeds: 85 2.4%

5%: hub to nonhub in seeds: 76, # of hubs in total: 336 22.6%

10%: nonhub to hub in roots: 3, # of hubs in roots: 326 0.9%

10%: hub to nonhub in roots: 143, # of hubs in total: 655 21.8%

10%: nonhub to hub in leaves: 13, # of hubs in leaves: 218 6.0%

10%: hub to nonhub in leaves: 123, # of hubs in total: 655 18.7%

10%: nonhub to hub in flowers: 18, # of hubs in flowers: 498 3.6%

10%: hub to nonhub in flowers: 62, # of hubs in total: 655 9.5%

10%: nonhub to hub in siliques: 17, # of hubs in siliques: 332 5.1%

10%: hub to nonhub in siliques: 109, # of hubs in total: 655 16.6%

10%: nonhub to hub in seeds: 10, # of hubs in seeds: 199 5.0%

10%: hub to nonhub in seeds: 98, # of hubs in total: 655 15.0%

20%: nonhub to hub in roots: 25, # of hubs in roots: 712 3.5%

20%: hub to nonhub in roots: 156, # of hubs in total: 1235 12.6%

20%: nonhub to hub in leaves: 25, # of hubs in leaves: 464 5.4%

20%: hub to nonhub in leaves: 162, # of hubs in total: 1235 13.1%

20%: nonhub to hub in flowers: 34, # of hubs in flowers: 938 3.6%

20%: hub to nonhub in flowers: 111, # of hubs in total: 1235 9.0%

20%: nonhub to hub in siliques: 27, # of hubs in siliques: 611 4.4%

20%: hub to nonhub in siliques: 180, # of hubs in total: 1235 14.6%

20%: nonhub to hub in seeds: 13, # of hubs in seeds: 344 3.8%

20%: hub to nonhub in seeds: 186, # of hubs in total: 1235 15.1%

For networks with 30% replaced interactions:

5%: nonhub to hub in roots: 2, # of hubs in roots: 113 1.8%

5%: hub to nonhub in roots: 67, # of hubs in total: 234 28.6%

5%: nonhub to hub in leaves: 7, # of hubs in leaves: 77 9.1%

5%: hub to nonhub in leaves: 51, # of hubs in total: 234 21.8%

5%: nonhub to hub in flowers: 6, # of hubs in flowers: 169 3.6%

5%: hub to nonhub in flowers: 33, # of hubs in total: 234 14.1%

5%: nonhub to hub in siliques: 2, # of hubs in siliques: 105 1.9%

5%: hub to nonhub in siliques: 49, # of hubs in total: 234 20.9%

5%: nonhub to hub in seeds: 4, # of hubs in seeds: 53 7.5%

5%: hub to nonhub in seeds: 62, # of hubs in total: 234 26.5%

10%: nonhub to hub in roots: 2, # of hubs in roots: 254 0.8%

10%: hub to nonhub in roots: 140, # of hubs in total: 538 26%

10%: nonhub to hub in leaves: 3, # of hubs in leaves: 155 1.9%

10%: hub to nonhub in leaves: 123, # of hubs in total: 538 22.9%

10%: nonhub to hub in flowers: 15, # of hubs in flowers: 390 3.8%

10%: hub to nonhub in flowers: 68, # of hubs in total: 538 12.6%

10%: nonhub to hub in siliques: 11, # of hubs in siliques: 245 4.5%

10%: hub to nonhub in siliques: 114, # of hubs in total: 538 21.2%

10%: nonhub to hub in seeds: 3, # of hubs in seeds: 142 2.1%

10%: hub to nonhub in seeds: 105, # of hubs in total: 538 19.5%

20%: nonhub to hub in roots: 1, # of hubs in roots: 592 0.2%

20%: hub to nonhub in roots: 257, # of hubs in total: 1231 20.9%

20%: nonhub to hub in leaves: 6, # of hubs in leaves: 348 1.7%

20%: hub to nonhub in leaves: 266, # of hubs in total: 1231 21.6%

20%: nonhub to hub in flowers: 0, # of hubs in flowers: 774 0%

20%: hub to nonhub in flowers: 250, # of hubs in total: 1231 20.3%

20%: nonhub to hub in siliques: 15, # of hubs in siliques: 579 2.6%

20%: hub to nonhub in siliques: 211, # of hubs in total: 1231 17.1%

20%: nonhub to hub in seeds: 11, # of hubs in seeds: 323 3.4%

20%: hub to nonhub in seeds: 216, # of hubs in total: 1231 17.5%

For networks with 40% replaced interactions:

5%: nonhub to hub in roots: 2, # of hubs in roots: 80 2.5%

5%: hub to nonhub in roots: 55, # of hubs in total: 179 30.7%

5%: nonhub to hub in leaves: 4, # of hubs in leaves: 51 7.8%

5%: hub to nonhub in leaves: 39, # of hubs in total: 179 21.8%

5%: nonhub to hub in flowers: 5, # of hubs in flowers: 119 4.2%

5%: hub to nonhub in flowers: 36, # of hubs in total: 179 20.1%

5%: nonhub to hub in siliques: 2, # of hubs in siliques: 69 2.9%

5%: hub to nonhub in siliques: 49, # of hubs in total: 179 27.4%

5%: nonhub to hub in seeds: 2, # of hubs in seeds: 39 5.1%

5%: hub to nonhub in seeds: 45, # of hubs in total: 179 25.1%

10%: nonhub to hub in roots: 1, # of hubs in roots: 203 0.5%

10%: hub to nonhub in roots: 133, # of hubs in total: 459 29.0%

10%: nonhub to hub in leaves: 4, # of hubs in leaves: 114 3.5%

10%: hub to nonhub in leaves: 127, # of hubs in total: 459 27.7%

10%: nonhub to hub in flowers: 13, # of hubs in flowers: 330 3.9%

10%: hub to nonhub in flowers: 66, # of hubs in total: 459 14.4%

10%: nonhub to hub in siliques: 11, # of hubs in siliques: 220 5%

10%: hub to nonhub in siliques: 97, # of hubs in total: 459 21.1%

10%: nonhub to hub in seeds: 7, # of hubs in seeds: 113 6.2%

10%: hub to nonhub in seeds: 106, # of hubs in total: 459 23.1%

20%: nonhub to hub in roots: 7, # of hubs in roots: 525 1.3%

20%: hub to nonhub in roots: 273, # of hubs in total: 1161 23.5%

20%: nonhub to hub in leaves: 7, # of hubs in leaves: 308 2.3%

20%: hub to nonhub in leaves: 278, # of hubs in total: 1161 23.9%

20%: nonhub to hub in flowers: 0, # of hubs in flowers: 694 0%

20%: hub to nonhub in flowers: 265, # of hubs in total: 1161 22.8%

20%: nonhub to hub in siliques: 15, # of hubs in siliques: 511 2.9%

20%: hub to nonhub in siliques: 226, # of hubs in total: 1161 19.5%

20%: nonhub to hub in seeds: 9, # of hubs in seeds: 299 3%

20%: hub to nonhub in seeds: 214, # of hubs in total: 1161 18.4%

For networks with 10% additional interactions:

5%: nonhub to hub in roots: 2, # of hubs in roots: 207 1.0%

5%: hub to nonhub in roots: 87, # of hubs in total: 399 21.8%

5%: nonhub to hub in leaves: 15, # of hubs in leaves: 142 10.6%

5%: hub to nonhub in leaves: 78, # of hubs in total: 399 19.5%

5%: nonhub to hub in flowers: 10, # of hubs in flowers: 297 3.4%

5%: hub to nonhub in flowers: 45, # of hubs in total: 399 11.3%

5%: nonhub to hub in siliques: 7, # of hubs in siliques: 188 3.7%

5%: hub to nonhub in siliques: 81, # of hubs in total: 399 20.3%

5%: nonhub to hub in seeds: 6, # of hubs in seeds: 110 5.5%

5%: hub to nonhub in seeds: 80, # of hubs in total: 399 20.1%

10%: nonhub to hub in roots: 1, # of hubs in roots: 379 0.3%

10%: hub to nonhub in roots: 154, # of hubs in total: 757 20.3%

10%: nonhub to hub in leaves: 8, # of hubs in leaves: 253 3.2%

10%: hub to nonhub in leaves: 140, # of hubs in total: 757 18.5%

10%: nonhub to hub in flowers: 21, # of hubs in flowers: 567 3.7%

10%: hub to nonhub in flowers: 79, # of hubs in total: 757 10.4%

10%: nonhub to hub in siliques: 19, # of hubs in siliques: 376 5.1%

10%: hub to nonhub in siliques: 121, # of hubs in total: 757 16.0%

10%: nonhub to hub in seeds: 12, # of hubs in seeds: 225 5.3%

10%: hub to nonhub in seeds: 110, # of hubs in total: 757 14.5%

20%: nonhub to hub in roots: 2, # of hubs in roots: 724 0.3%

20%: hub to nonhub in roots: 227, # of hubs in total: 1420 16.0%

20%: nonhub to hub in leaves: 3, # of hubs in leaves: 466 0.6%

20%: hub to nonhub in leaves: 225, # of hubs in total: 1420 15.8%

20%: nonhub to hub in flowers: 0, # of hubs in flowers: 986 0%

20%: hub to nonhub in flowers: 183, # of hubs in total: 1420 12.9%

20%: nonhub to hub in siliques: 24, # of hubs in siliques: 710 3.4%

20%: hub to nonhub in siliques: 187, # of hubs in total: 1420 13.2%

20%: nonhub to hub in seeds: 14, # of hubs in seeds: 417 3.4%

20%: hub to nonhub in seeds: 193, # of hubs in total: 1420 13.6%

For networks with 20% additional interactions:

5%: nonhub to hub in roots: 0, # of hubs in roots: 213 0%

5%: hub to nonhub in roots: 98, # of hubs in total: 426 23.0%

5%: nonhub to hub in leaves: 17, # of hubs in leaves: 157 10.8%

5%: hub to nonhub in leaves: 74, # of hubs in total: 426 17.4%

5%: nonhub to hub in flowers: 7, # of hubs in flowers: 308 2.3%

5%: hub to nonhub in flowers: 56, # of hubs in total: 426 13.1%

5%: nonhub to hub in siliques: 5, # of hubs in siliques: 195 2.6%

5%: hub to nonhub in siliques: 89, # of hubs in total: 426 20.9%

5%: nonhub to hub in seeds: 4, # of hubs in seeds: 118 3.4%

5%: hub to nonhub in seeds: 85, # of hubs in total: 426 20.0%

10%: nonhub to hub in roots: 1, # of hubs in roots: 399 0.3%

10%: hub to nonhub in roots: 164, # of hubs in total: 802 20.4%

10%: nonhub to hub in leaves: 5, # of hubs in leaves: 260 1.9%

10%: hub to nonhub in leaves: 151, # of hubs in total: 802 18.8%

10%: nonhub to hub in flowers: 21, # of hubs in flowers: 593 3.5%

10%: hub to nonhub in flowers: 86, # of hubs in total: 802 10.7%

10%: nonhub to hub in siliques: 12, # of hubs in siliques: 395 3.0%

10%: hub to nonhub in siliques: 116, # of hubs in total: 802 14.5%

10%: nonhub to hub in seeds: 9, # of hubs in seeds: 232 3.9%

10%: hub to nonhub in seeds: 116, # of hubs in total: 802 14.5%

20%: nonhub to hub in roots: 5, # of hubs in roots: 773 0.6%

20%: hub to nonhub in roots: 269, # of hubs in total: 1541 17.5%

20%: nonhub to hub in leaves: 7, # of hubs in leaves: 489 1.4%

20%: hub to nonhub in leaves: 259, # of hubs in total: 1541 16.8%

20%: nonhub to hub in flowers: 0, # of hubs in flowers: 1039 0%

20%: hub to nonhub in flowers: 236, # of hubs in total: 1541 15.3%

20%: nonhub to hub in siliques: 19, # of hubs in siliques: 755 2.5%

20%: hub to nonhub in siliques: 222, # of hubs in total: 1541 14.4%

20%: nonhub to hub in seeds: 8, # of hubs in seeds: 431 1.9%

20%: hub to nonhub in seeds: 233, # of hubs in total: 1541 15.1%

***Network statistics with additional interactions simulating false negatives***

| **network** | **node#** | **edge#** | **avg.de** | **max.de** | **avg.cc** | **avg.spl** | **max.spl** | **avg.ecc** | **avg.be** | **max.be** |
| --- | --- | --- | --- | --- | --- | --- | --- | --- | --- | --- |
| total_20pfn | 6606 | 26598 | 8.05 | 172 | 0.22 | 4.47 | 15 | 9.47 | 8,608.85 | 732,669.14 |
| rand_total_20pfn | 5899 | 26598 | 9.02 | 148 | 0.01 | 3.81 | 11 | 7.13 | 8,237.98 | 468,120.21 |
| roots_20pfn | 3318 | 12137 | 7.32 | 128 | 0.22 | 4.25 | 11 | 7.09 | 4,186.40 | 280,372.46 |
| rand_roots_20pfn | 3146 | 11679 | 7.42 | 99 | 0.02 | 3.84 | 10 | 7.08 | 4,436.92 | 181,072.63 |
| leaves_20pfn | 2305 | 6520 | 5.66 | 102 | 0.21 | 4.4 | 12 | 7.3 | 2,776.13 | 204,238.02 |
| rand_leaves_20pfn | 2098 | 5862 | 5.59 | 64 | 0.01 | 4.09 | 10 | 7.11 | 3,146.20 | 111,455.17 |
| flowers_20pfn | 4683 | 17626 | 7.53 | 152 | 0.22 | 4.4 | 12 | 7.82 | 6,135.64 | 485,416.01 |
| rand_flowers_20pfn | 4293 | 17319 | 8.07 | 119 | 0.01 | 3.85 | 9 | 6.91 | 6,087.00 | 290,136.18 |
| siliques_20pfn | 3093 | 10350 | 6.69 | 79 | 0.23 | 4.33 | 12 | 7.41 | 3,850.60 | 125,301.80 |
| rand_siliques_20pfn | 2887 | 9704 | 6.72 | 78 | 0.01 | 3.96 | 10 | 7.45 | 4,223.76 | 149,686.59 |
| seeds_20pfn | 1869 | 5161 | 5.52 | 61 | 0.22 | 4.38 | 12 | 7.53 | 2,263.76 | 59,188.99 |
| rand_seeds_20pfn | 1733 | 4581 | 5.29 | 57 | 0.02 | 4.12 | 9 | 6.96 | 2,634.37 | 85,431.54 |

Avg.de: average degree; Max.de: maximum degree; Avg.cc: average clustering coefficient; Avg.spl: average shortest path length; Avg.ecc: average eccentricity; Avg.be: average betweenness; Max.be: maximum betweenness.
