# Supplementary material for: Investigating the validity of current network analysis on static conglomerate networks by protein network stratification
Source: BMC Bioinformatics. 2010 Sep 16;11:466. doi: 10.1186/1471-2105-11-466 (PMC2949894; doi:10.1186/1471-2105-11-466)
Supplement: Additional file 5 — Results of analyses using human PPI networks. When using human PPI networks for analysis, results showed general consistency with the results based on A. thaliana networks. Network statistics including degree, clustering coefficient, eccentricity and betweenness were calculated and compared. Network compatibility was assessed. Network hub/bottleneck status changes were also investigated. [file 1471-2105-11-466-S5.DOC]

**Additional file 5**

**Preliminary Network Analysis Using Human PPI Networks**

***Network Statistics are Different between Conglomerate Networks and Brain/Kidney Subnetworks***

| Degree | hkupp_cong | hkupp_hprd | hubpp_cong | hubpp_hprd |
| --- | --- | --- | --- | --- |
| hkupp_cong | 1 | 3.37E-27 | 6.55E-11 | 3.46E-30 |
| hkupp_hprd | 3.37E-27 | 1 | 1.09E-06 | 0.124454 |
| hubpp_cong | 6.55E-11 | 1.09E-06 | 1 | 3.94E-09 |
| hubpp_hprd | 3.46E-30 | 0.124454 | 3.94E-09 | 1 |
|  |  |  |  |  |
|  |  |  |  |  |
| Clustering Co. | hkupp_cong | hkupp_hprd | hubpp_cong | hubpp_hprd |
| hkupp_cong | 1 | 1.21E-50 | 0.389567 | 2.19E-32 |
| hkupp_hprd | 1.21E-50 | 1 | 4.30E-38 | 0.198272 |
| hubpp_cong | 0.389567 | 4.30E-38 | 1 | 1.59E-27 |
| hubpp_hprd | 2.19E-32 | 0.198272 | 1.59E-27 | 1 |
|  |  |  |  |  |
|  |  |  |  |  |
| Eccentricity | hkupp_cong | hkupp_hprd | hubpp_cong | hubpp_hprd |
| hkupp_cong | 1 | 1.00E-49 | 8.79E-07 | 0.325359 |
| hkupp_hprd | 1.00E-49 | 1 | 1.48E-47 | 6.96E-20 |
| hubpp_cong | 8.79E-07 | 1.48E-47 | 1 | 1.74E-06 |
| hubpp_hprd | 0.325359 | 6.96E-20 | 1.74E-06 | 1 |
|  |  |  |  |  |
|  |  |  |  |  |
| Betweenness | hkupp_cong | hkupp_hprd | hubpp_cong | hubpp_hprd |
| hkupp_cong | 1 | 0.121298 | 1.11E-09 | 1.45E-13 |
| hkupp_hprd | 0.121298 | 1 | 0.000112 | 3.51E-07 |
| hubpp_cong | 1.11E-09 | 0.000112 | 1 | 0.094313 |
| hubpp_hprd | 1.45E-13 | 3.51E-07 | 0.094313 | 1 |

Hkupp_cong is a kidney subnetwork stratified from a pooled conglomerate human PPI network [1]. Hubpp_cong is a brain subnetwork stratified from the pooled conglomerate human PPI network. Hkupp_hprd is a kidney subnetwork stratified from a conglomerate human PPI network using all interactions from the seventh version of HPRD database [2]. Hubpp_hprd is a brain subnetwork stratified from a conglomerate human PPI network using all interactions from HPRD database.

***Summary of Compatibility between Stratified Human Kidney and Brain Subnetworks***

|  |  | node overlap | Jaccard Index | interaction overlap | Jaccard Index |
| --- | --- | --- | --- | --- | --- |
| hkupp_gene_list | hubpp_gene_list | 845 | 0.232 | na | na |
| 2949 nodes | 1536 nodes |  |  |  |  |
|  |  |  |  |  |  |
| hkupp_cong.dat | hubpp_chuangset.dat | 575 | 0.294 | 1418 | 0.183 |
| 1593 nodes/ 6498 ints | 937 nodes/ 2674 ints |  |  |  |  |
|  |  |  |  |  |  |
| hkupp_hprd2.dat | hubpp_hprd.dat |  |  |  |  |
| 1417 nodes/ 3117 ints | 803 nodes/ 1616 ints | 475 | 0.272 | 727 | 0.181 |

***Hub and Bottleneck Status Change Using Human PPI Networks***

Hub Status Change Using Human PPI Networks

| 5%: nonhub to hub in kidney_cong: 0, # of hubs in kidney_cong: 79 | 0% |
| --- | --- |
| 5%: hub to nonhub in kidney_cong: 134, # of hubs in cong: 565 | 23.70% |
| 5%: nonhub to hub in brain_cong: 0, # of hubs in brain_ccong: 46 | 0% |
| 5%: hub to nonhub in brain_cong: 81, # of hubs in cong: 565 | 14.30% |
| 5%: nonhub to hub in kidney_hprd: 2, # of hubs in kidney_hprd: 69 | 2.90% |
| 5%: hub to nonhub in kidney_hprd: 96, # of hubs in hprd: 482 | 19.90% |
| 5%: nonhub to hub in brain_hprd: 2, # of hubs in brain_hprd: 44 | 4.50% |
| 5%: hub to nonhub in brain_hprd: 65, # of hubs in hprd: 482 | 13.50% |
|  |  |
| 10%: nonhub to hub in kidney_cong: 0, # of hubs in kidney_cong: 158 | 0% |
| 10%: hub to nonhub in kidney_cong: 218, # of hubs in cong: 1105 | 19.70% |
| 10%: nonhub to hub in brain_cong: 0, # of hubs in brain_ccong: 95 | 0% |
| 10%: hub to nonhub in brain_cong: 134, # of hubs in cong: 1105 | 12.10% |
| 10%: nonhub to hub in kidney_hprd: 3, # of hubs in kidney_hprd: 137 | 2.20% |
| 10%: hub to nonhub in kidney_hprd: 176, # of hubs in hprd: 961 | 18.30% |
| 10%: nonhub to hub in brain_hprd: 0, # of hubs in brain_hprd: 76 | 0% |
| 10%: hub to nonhub in brain_hprd: 114, # of hubs in hprd: 961 | 11.90% |
|  |  |
| 20%: nonhub to hub in kidney_cong: 0, # of hubs in kidney_cong: 305 | 0% |
| 20%: hub to nonhub in kidney_cong: 377, # of hubs in cong: 2172 | 17.40% |
| 20%: nonhub to hub in brain_cong: 4, # of hubs in brain_ccong: 195 | 2.10% |
| 20%: hub to nonhub in brain_cong: 211, # of hubs in cong: 2172 | 9.70% |
| 20%: nonhub to hub in kidney_hprd: 6, # of hubs in kidney_hprd: 253 | 2.40% |
| 20%: hub to nonhub in kidney_hprd: 305, # of hubs in hprd: 1912 | 16.00% |
| 20%: nonhub to hub in brain_hprd: 4, # of hubs in brain_hprd: 155 | 2.60% |
| 20%: hub to nonhub in brain_hprd: 189, # of hubs in hprd: 1912 | 9.90% |

Kidney_cong is a kidney subnetwork stratified from a pooled conglomerate human PPI network [1]. Brain_cong is a brain subnetwork stratified from the pooled conglomerate human PPI network. Kidney _hprd is a kidney subnetwork stratified from a conglomerate human PPI network using all interactions from the seventh version of HPRD database [2]. Brain _hprd is a brain subnetwork stratified from a conglomerate human PPI network using all interactions from HPRD database.

Bottleneck Status Change Using Human PPI Networks

| 5%: nonbott to bott in kidney_cong: 8, # of botts in kidney_cong: 80 | 10% |
| --- | --- |
| 5%: bott to nonbott in kidney_cong: 139, # of botts in cong: 560 | 24.80% |
| 5%: nonbott to bott in brain_cong: 7, # of botts in brain_ccong: 47 | 14.90% |
| 5%: bott to nonbott in brain_cong: 102, # of botts in cong: 560 | 18.20% |
| 5%: nonbott to bott in kidney_hprd: 4, # of botts in kidney_hprd: 71 | 5.60% |
| 5%: bott to nonbott in kidney_hprd: 101, # of botts in hprd: 465 | 21.70% |
| 5%: nonbott to bott in brain_hprd: 2, # of botts in brain_hprd: 40 | 5% |
| 5%: bott to nonbott in brain_hprd: 76, # of botts in hprd: 465 | 16.30% |
|  |  |
| 10%: nonbott to bott in kidney_cong: 18, # of botts in kidney_cong: 159 | 11.30% |
| 10%: bott to nonbott in kidney_cong: 239, # of botts in cong: 1120 | 21.30% |
| 10%: nonbott to bott in brain_cong: 17, # of botts in brain_ccong: 94 | 18.10% |
| 10%: bott to nonbott in brain_cong: 164, # of botts in cong: 1120 | 14.60% |
| 10%: nonbott to bott in kidney_hprd: 12, # of botts in kidney_hprd: 142 | 8.50% |
| 10%: bott to nonbott in kidney_hprd: 178, # of botts in hprd: 931 | 19.10% |
| 10%: nonbott to bott in brain_hprd: 12, # of botts in brain_hprd: 80 | 15% |
| 10%: bott to nonbott in brain_hprd: 125, # of botts in hprd: 931 | 13.40% |
|  |  |
| 20%: nonbott to bott in kidney_cong: 41, # of botts in kidney_cong: 319 | 12.90% |
| 20%: bott to nonbott in kidney_cong: 381, # of botts in cong: 2241 | 17% |
| 20%: nonbott to bott in brain_cong: 22, # of botts in brain_ccong: 187 | 11.80% |
| 20%: bott to nonbott in brain_cong: 247, # of botts in cong: 2241 | 11.00% |
| 20%: nonbott to bott in kidney_hprd: 24, # of botts in kidney_hprd: 283 | 8.50% |
| 20%: bott to nonbott in kidney_hprd: 273, # of botts in hprd: 1861 | 14.70% |
| 20%: nonbott to bott in brain_hprd: 24, # of botts in brain_hprd: 162 | 14.80% |
| 20%: bott to nonbott in brain_hprd: 205, # of botts in hprd: 1861 | 11.00% |

Kidney_cong is a kidney subnetwork stratified from a pooled conglomerate human PPI network [1]. Brain_cong is a brain subnetwork stratified from the pooled conglomerate human PPI network. Kidney _hprd is a kidney subnetwork stratified from a conglomerate human PPI network using all interactions from the seventh version of HPRD database [2]. Brain _hprd is a brain subnetwork stratified from a conglomerate human PPI network using all interactions from HPRD database.

**References**

1. Chuang HY, Lee E, Liu YT, Lee D, Ideker T: **Network-based classification of breast cancer metastasis.** *Mol Syst Biol* 2007, **3**: 140.

2. Ramani AK, Bunescu RC, Mooney RJ, Marcotte EM: **Consolidating the set of known human protein-protein interactions in preparation for large-scale mapping of the human interactome.** *Genome Biol* 2005, **6**(5): R40.
